# Long non-coding RNA expression patterns in lung tissues of chronic cigarette smoke induced COPD mouse model

**DOI:** 10.1038/s41598-018-25702-3

**Published:** 2018-05-15

**Authors:** Haiyun Zhang, Dejun Sun, Defu Li, Zeguang Zheng, Jingyi Xu, Xue Liang, Chenting Zhang, Sheng Wang, Jian Wang, Wenju Lu

**Affiliations:** 1 State Key Laboratory of Respiratory Diseases, Guangzhou Institute of Respiratory Health, The First Affiliated Hospital of Guangzhou Medical University, Guangzhou, Guangdong 510180 China; 2 Division of Translational and Regenerative Medicine, Department of Medicine, The University of Arizona, Tucson, AZ 85721-0202 USA; 3 Division of Pulmonary Medicine, The People’s Hospital of Inner Mongolia, Inner Mongolia Medical University, Hohhot, Inner Mongolia China

**Keywords:** Gene expression, Chronic obstructive pulmonary disease

## Abstract

Long non-coding RNAs (lncRNAs) have critical regulatory roles in protein-coding gene expression. Aberrant expression profiles of lncRNAs have been observed in various human diseases. In this study, we investigated transcriptome profiles in lung tissues of chronic cigarette smoke (CS)-induced COPD mouse model. We found that 109 lncRNAs and 260 mRNAs were significantly differential expressed in lungs of chronic CS-induced COPD mouse model compared with control animals. GO and KEGG analyses indicated that differentially expressed lncRNAs associated protein-coding genes were mainly involved in protein processing of endoplasmic reticulum pathway, and taurine and hypotaurine metabolism pathway. The combination of high throughput data analysis and the results of qRT-PCR validation in lungs of chronic CS-induced COPD mouse model, 16HBE cells with CSE treatment and PBMC from patients with COPD revealed that NR_102714 and its associated protein-coding gene UCHL1 might be involved in the development of COPD both in mouse and human. In conclusion, our study demonstrated that aberrant expression profiles of lncRNAs and mRNAs existed in lungs of chronic CS-induced COPD mouse model. From animal models perspective, these results might provide further clues to investigate biological functions of lncRNAs and their potential target protein-coding genes in the pathogenesis of COPD.

## Introduction

Chronic Obstructive Lung Disease (COPD) is a common, preventable, and treatable respiratory disease^1^. It has become a global health problem, accounting for severe mortality and disease burden all over the world^2^. Cigarette smoke (CS) exposure is one of the most significant risk factors associated with COPD, which causes persistent airflow limitation and enhanced chronic inflammatory response within airways^1,3^. An estimated 90% of all deaths from COPD are attributed to CS^4^. CS comprises more than 4,500 chemical compounds and over 10^15^ oxidants in its gaseous and particulate phases. Many of these oxidants are relatively long-lived^5^. CS-induced oxidants produce high level of reactive oxygen species (ROS) by activating inflammatory cells and airway epithelial cells^6^. It has been demonstrated excessive ROS probably leads to great level of oxidative stress in cigarette smokers. Excessive ROS causes lung tissue damage by inhibiting antiprotease processes, and accelerating the macro-molecular degradation such as DNA, proteins, and other cell components^7^. High level of ROS also postpones the resolution of inflammation by injurying phagocytosis of alveolar macrophages, and therefore contributes to autophagy-impairment and emphysema^8^. Moreover, high level of ROS may lead to lung fibrotic remodeling via impairing alveolar epithelial type II cells repetitively, promoting the formation of epithelial-to-mesenchymal transition (EMT), and accelerating the transition of fibroblasts into aggressive myofibroblast^9^. Considering these aspects, CS-induced oxidative stress may in part account for the initiation and progression of COPD. It appears that better control of CS-induced oxidative stress via regulating critical molecules in the generation- and metabolism- pathways of ROS, might serve as a promising therapeutic strategy for COPD.

Long non-coding RNAs (lncRNAs) are a kind of RNAs with lengths above 200 nucleotides, whose protein-coding capabilities are inferior to that of mRNAs. Usually, lncRNAs are classified into 5 categories on the basis of their locations relative to nearby protein-coding genes: (1) sense lncRNAs; (2) antisense lncRNAs; (3) bidirectional lncRNAs; (4) intronic lncRNAs; (5) intergenic lncRNAs^10^. LncRNAs regulate protein-coding genes expression through epigenetic^11^, transcriptional^12^ or post-transcriptional^13^ patterns. Their superior tissue-specific expression manners over protein-coding genes make them become attractive diagnostic and therapeutic targets in the future^14^. Recently, emerging evidences have suggested that aberrant expression of lncRNAs is observed in various human diseases, including COPD. Some lncRNAs have been determined to have biological functions in the pathogenesis of COPD, such as smoke and cancer-associated lncRNA-1 (SCAL1).SCAL1 is high expressed in the airway epithelia of cigarette smokers versus non-smokers. The expression level of SCAL1 is elevated in numerous lung cancer cell lines after CS extract treatment. Knockdown of SCAL1 results in increased CS-induced toxicity, suggesting that SCAL1 protects lung cells against CS-induced oxidative stress^15^. However, many lncRNAs and their biological functions involved in COPD are still unclear. RNA sequencing is a useful technology with an advantage in discovering novel tissue-and disease-specific ncRNAs, whereas microarray and qRT-PCR can only measure the expression levels of known ncRNAs^10,16^. To date, RNA sequencing has been widely used in transcriptome profiles analyses^17^.

In the present study, we used RNA sequencing to compare the expression profiles of lncRNAs and mRNAs in lungs of chronic CS-induced COPD mouse model with that of control animals. Next, we performed bioinformatics analyses to predict the gene sets of differentially expressed lncRNAs associated protein-coding genes, and further analyzed their biological functions. Therefore, from animal models perspective, this study would enhance our understanding of the different transcriptome profiles between lungs of chronic CS-induced COPD mouse model and that of control animals, which might provide further clues for future investigation of the biological functions of lncRNAs and their associated protein-coding genes in the onset and progression of COPD.

## Results

### Evaluation of chronic CS-induced COPD mouse model

**Figure 1:**
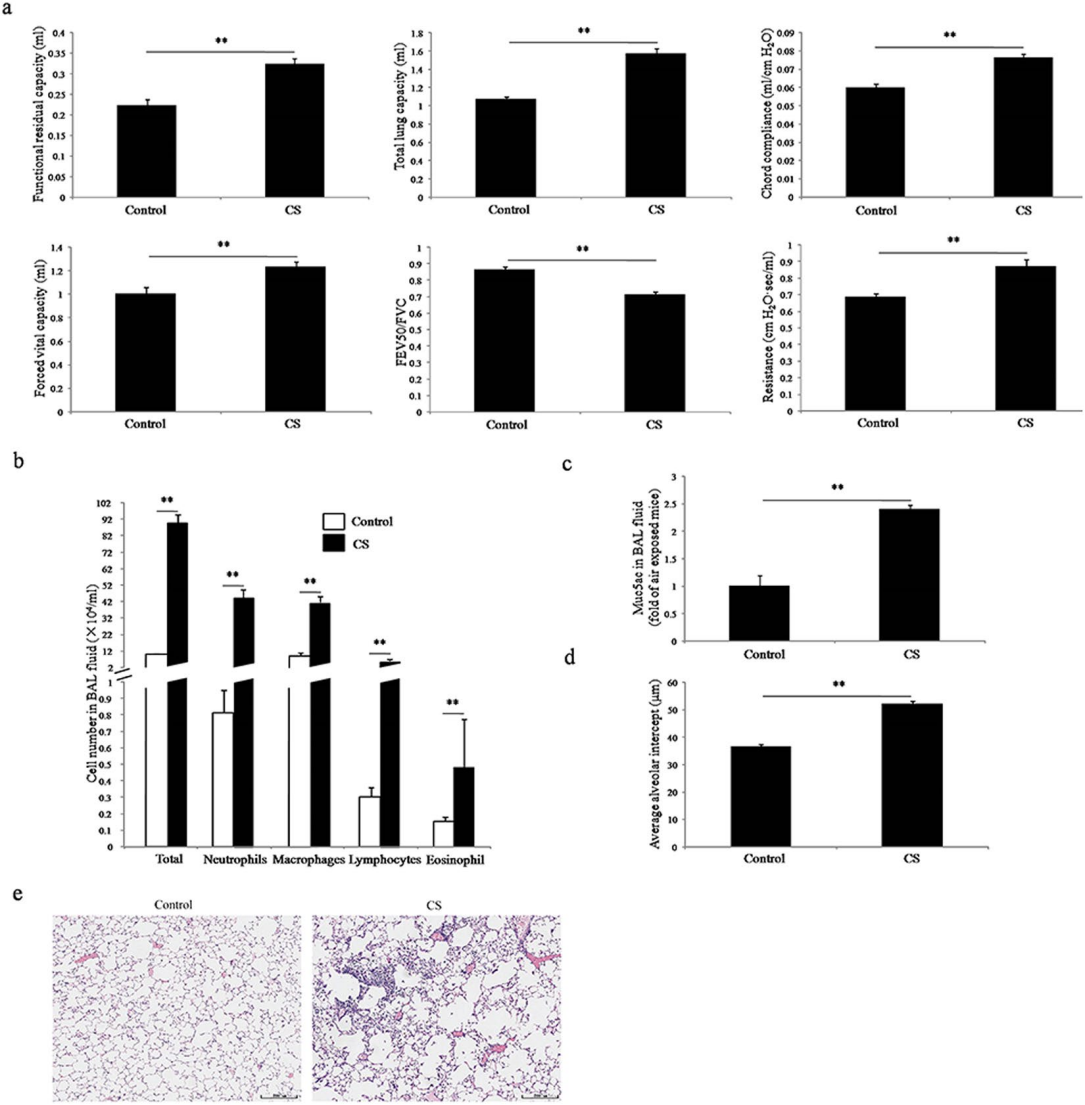
Evaluation of chronic CS-induced COPD mouse model. (**a**) Pulmonary function, (**b**) classification and counting of the cells in BAL fluid, (**c**) muc5AC in BAL fluid, (**d**) average alveolar intercept and (**e**) lung tissue morphology are examined in chronic CS-induced COPD mouse model and control animals. Control and CS indicate control animals and chronic CS-induced COPD model mice, respectively (n = 8, *p < 0.05, **p < 0.01).

Compared to control animals, the chronic CS exposed mouse model exhibited impaired lung function, mucus hypersecretion, thickened alveolar walls, enlarged alveolar area, loss of bronchial epithelial cilia, and increased inflammatory cells infiltration in both bronchoalveolar lavage (BAL) fluid and lung interstitial (Fig. 1). These results suggested that chronic CS-induced COPD mouse model was successfully established.

### Evaluation of RNA sequencing data

In raw reads of each sample, the percentages of adaptors, reads containing N, low quality reads (whose poor-quality bases inside were more than 20%), reads containing ribosome RNA, other unqualified reads (e.g., reads without 3′ adapters, reads without judgmental sequences, reads with 5′ adaptors contamination), and the effective reads were calculated (Supplementary Table S1 and Supplementary Figure S1a). The secondary RNA sequencing data analysis included mapping of the effective reads to mouse reference genome (Supplementary Table S2). More than 80% of raw reads in each sample were the effective reads, which were high quality data and could be used in subsequent sequence alignment analyses on genes and genomes. Distributions of the effective reads on the location of genes and genomes were shown in Supplementary Fig. S1b,c respectively. These results suggested that the RNA sequencing data of each sample satisfied sequencing requirement, and were able to be used in subsequent bioinformatics analyses.

### Overviews of differential expressed lncRNAs and mRNAs in lungs of chronic CS-induced COPD mouse model versus control animals

Unsupervised hierarchical clustering analysis clearly indicated that lungs of chronic CS-induced COPD mouse model exhibited obviously different expression profiles of lncRNAs and mRNAs compared to that of control animals (Supplementary Figure S2).Volcano plots depicted statistic significances and differential expression levels of lncRNAs and mRNAs between lungs of chronic CS-induced COPD mouse model and control animals (Supplementary Figure S3).

### Differentially expressed lncRNAs in lungs of chronic CS-induced COPD mouse model

37,072 lncRNAs were detected in lungs of chronic CS-induced COPD mouse model and control animals. 109 lncRNAs were significantly differential expressed between these two groups (fold change >2; padj<0.05). Among these lncRNAs, 51 lncRNAs were significantly up-regulated in chronic CS-induced COPD mouse model versus control animals, whereas 58 lncRNAs were significantly down-regulated. Top 20 significantly up- and down- regulated lncRNAs were summarized in Supplementary TableS3.

### Differentially expressed mRNAs in lungs of chronic CS-induced COPD mouse model

Similarly, 27,457 mRNAs were found between chronic CS-induced COPD mouse model and control animals. 260 mRNAs were significantly differential expressed between these two groups (fold change >2; padj<0.05). Among these mRNAs, 132 mRNAs were significantly up-regulated in chronic CS-induced COPD mouse model versus control animals, whereas 175 mRNAs were significantly down-regulated. Top 20 significantly up- and down- regulated mRNAs were provided in Supplementary TableS4.

### Prediction of significantly differential expressed lncRNAs associated protein-coding genes

Significantly differential expressed lncRNAs associated protein-coding genes were predicted via cis- and trans- regulation analyses respectively. In cis-regulation analysis, 93 significantly differential expressed lncRNAs, 89 lncRNAs potential target protein-coding genes, as well as 126 connections between these lncRNAs and their associated protein-coding genes were identified. In trans-regulation analysis, 63 significantly differential expressed lncRNAs, 810 lncRNAs potential target protein-coding genes, as well as 1890 connections between these lncRNAs and their associated protein-coding genes were identified. Combined the results of cis- and trans- regulation analyses, there were overlaps of 56 significantly differential expressed lncRNAs, 28 lncRNA potential target protein-coding genes, and 41 connections between these lncRNAs and their associated protein-coding genes (Supplementary Figure S4).

### Gene ontology (GO), Kyoto Encyclopedia of Genes and Genomes (KEGG) and Chemicals enrichment analyses of lncRNA potential target protein-coding genes

**Figure 2:**
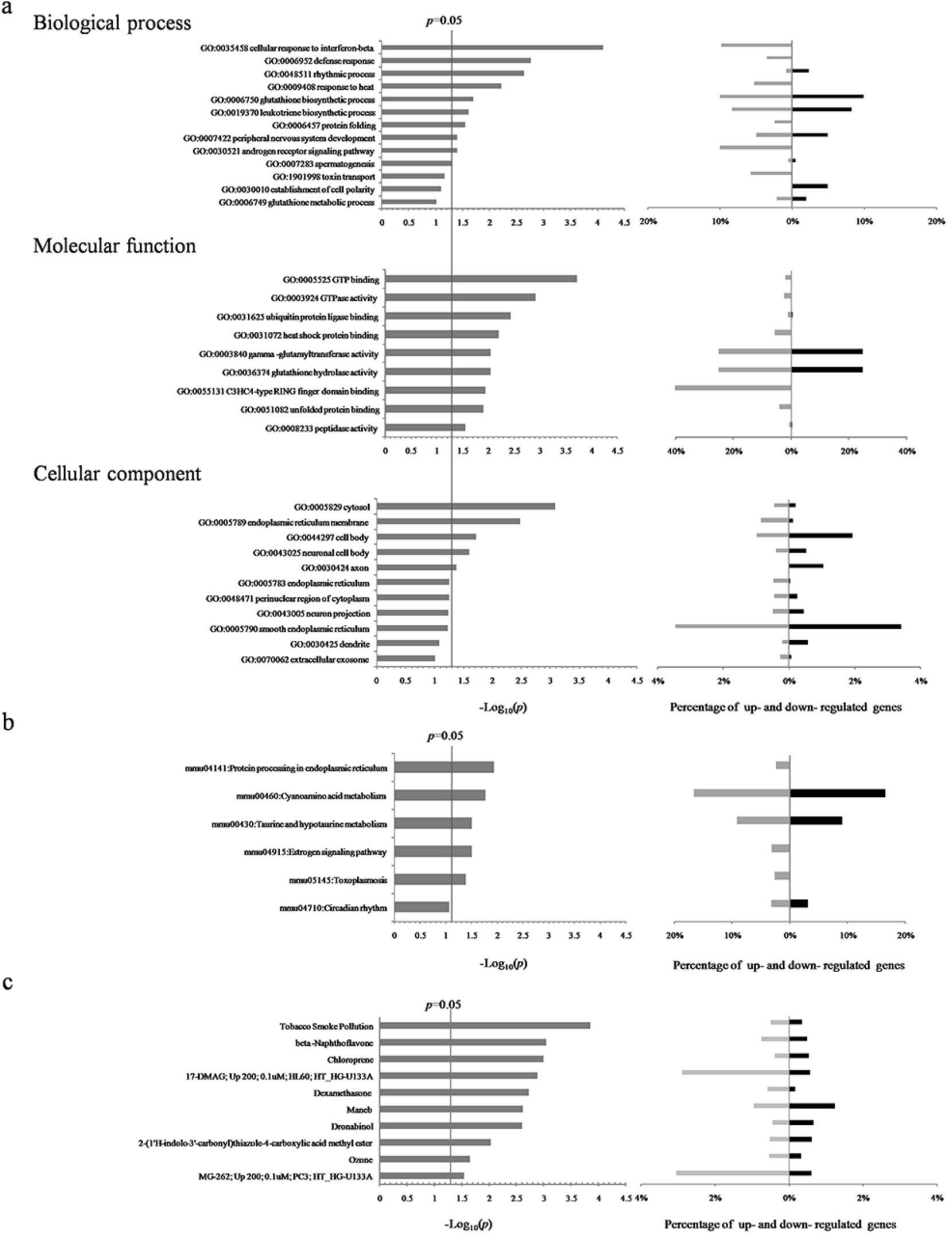
GO, KEGG and Chemicals enrichment analyses for the differential expression profile of lncRNA potential target protein-coding genes between chronic CS-induced COPD model mice and control animals. (**a**) In GO analysis, left picture shows statistic significances of perturbed GO nodes in molecular function, molecular function and cellular component. Right picture shows percentages of up-regulated genes (black bars, on the right side) and down-regulated genes (gray bars, on the left side) in these perturbed GO nodes. (**b**) In KEGG pathway analysis, left picture shows statistic significances of perturbed KEGG pathways. Both left picture and right picture of (b) KEGG pathway analysis and (**c**) Chemicals enrichment analysis have the same meaning as in (a).

For practical purpose, 871 lncRNAs potential target protein-coding genes were integrated with 260 significantly differential expressed mRNAs to narrow down the field of these protein-coding genes for enrichment analyses. Totally, 44 protein-coding genes were found. Biological process, molecular function, and cellular component of GO enrichment analyses for these protein-coding genes were shown in Fig. 2a. GO nodes with the highest statistic significances were cellular response to interferon-beta (biological process, GO: 0035458, 41 gene products), GTP binding (molecular function, GO: 0005525, 383 gene products) and cytosol (cellular component, GO: 0005829, 1784 gene products). KEGG pathway analysis suggested protein processing in endoplasmic reticulum (ER) to be the most significantly altered pathway in chronic CS-induced COPD mouse model versus control animals (mmu04141, 168 gene products; see Fig. 2b). Another significantly perturbed KEGG pathway was taurine and hypotaurine metabolism pathway (mmu00430, 11 gene products; see Fig. 2b). Chemicals enrichment analysis suggested that tobacco smoke pollution was most likely to be responsible for aberrant expression profiles of lncRNAs and mRNAs (Fig. 2c).

### Co-expression network of significantly differential expressed lncRNAs and their associated protein-coding genes

**Figure 3:**
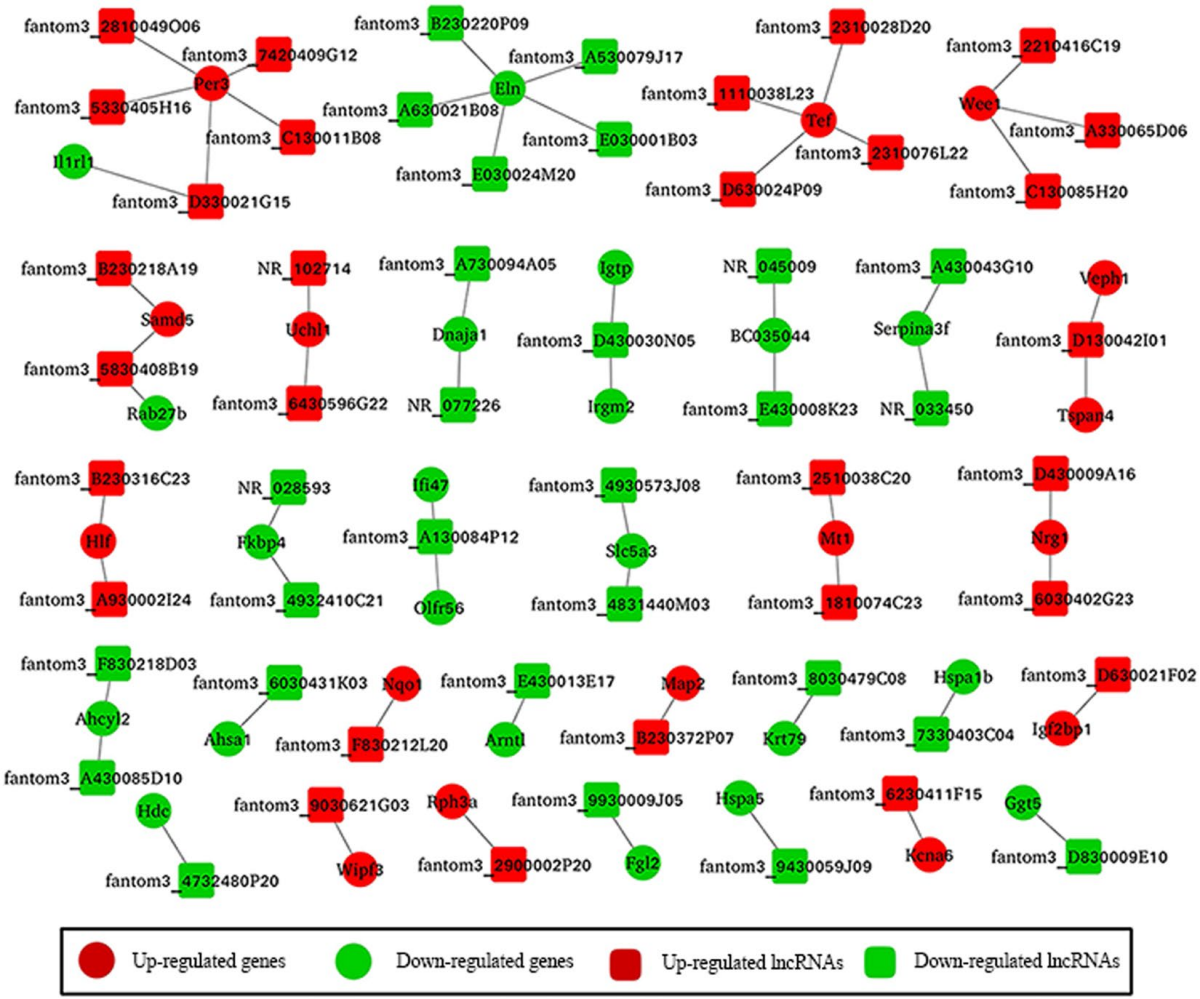
Co-expression network of significantly differential expressed lncRNAs and their associated protein-coding genes.

Base on the correlation between lncRNAs and their associated protein-coding genes, 56 significantly differential expressed lncRNAs and 37 protein-coding genes were selected to construct a co-expression network (inclusion criteria, pearson correlation coefficients >0.95). It was composed of 93 network nodes and 61 connections between 56 lncRNAs and 37 protein-coding genes. Within this network, one lncRNA could target 2 protein-coding genes at most, and one protein-coding gene could be associated with 5 lncRNAs at most (Fig. 3).

### An overlap of significantly differential expressed lncRNAs associated protein-coding genes between COPD mouse model and patients with COPD

**Figure 4:**
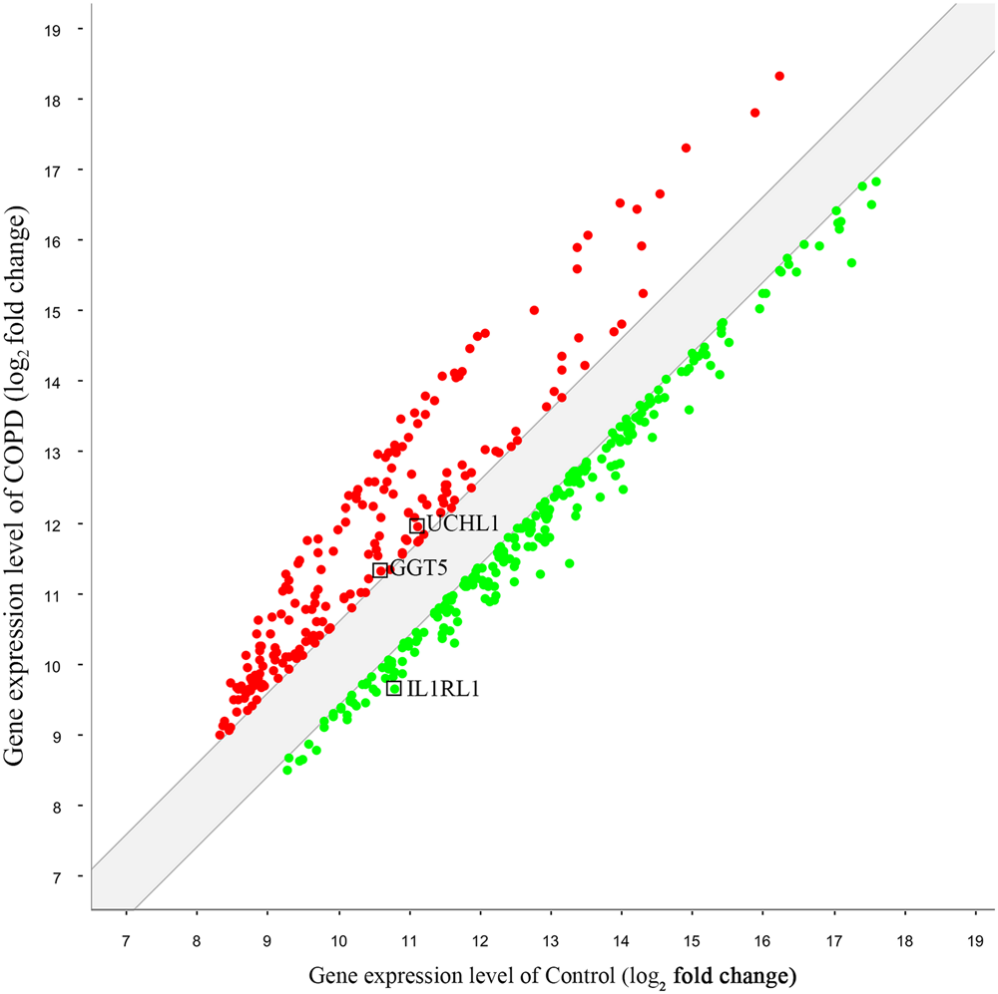
Significantly differential expressed protein-coding genes between chronic CS-induced COPD mouse model and patients with COPD.

Base on GEO datasets, 16 high throughput studies about patients with COPD were selected (Supplementary Table S5). A total of 525 significantly differential expressed protein-coding genes between patients with COPD and healthy control subjects were found. Next, these genes were overlapped with 44 differential expressed lncRNAs associated protein-coding genes, which were found in COPD mouse model versus control animals in the present study. As a result, IL1RL1, UCHL1 and GGT5 were the common significantly differential expressed protein-coding genes in both patients with COPD and chronic CS-induced COPD mouse model when compared to control subjects (Fig. 4).

### Quantitative RT-PCR (qRT-PCR) validation of significantly differential expressed lncRNAs and mRNAs in mouse

**Figure 5:**
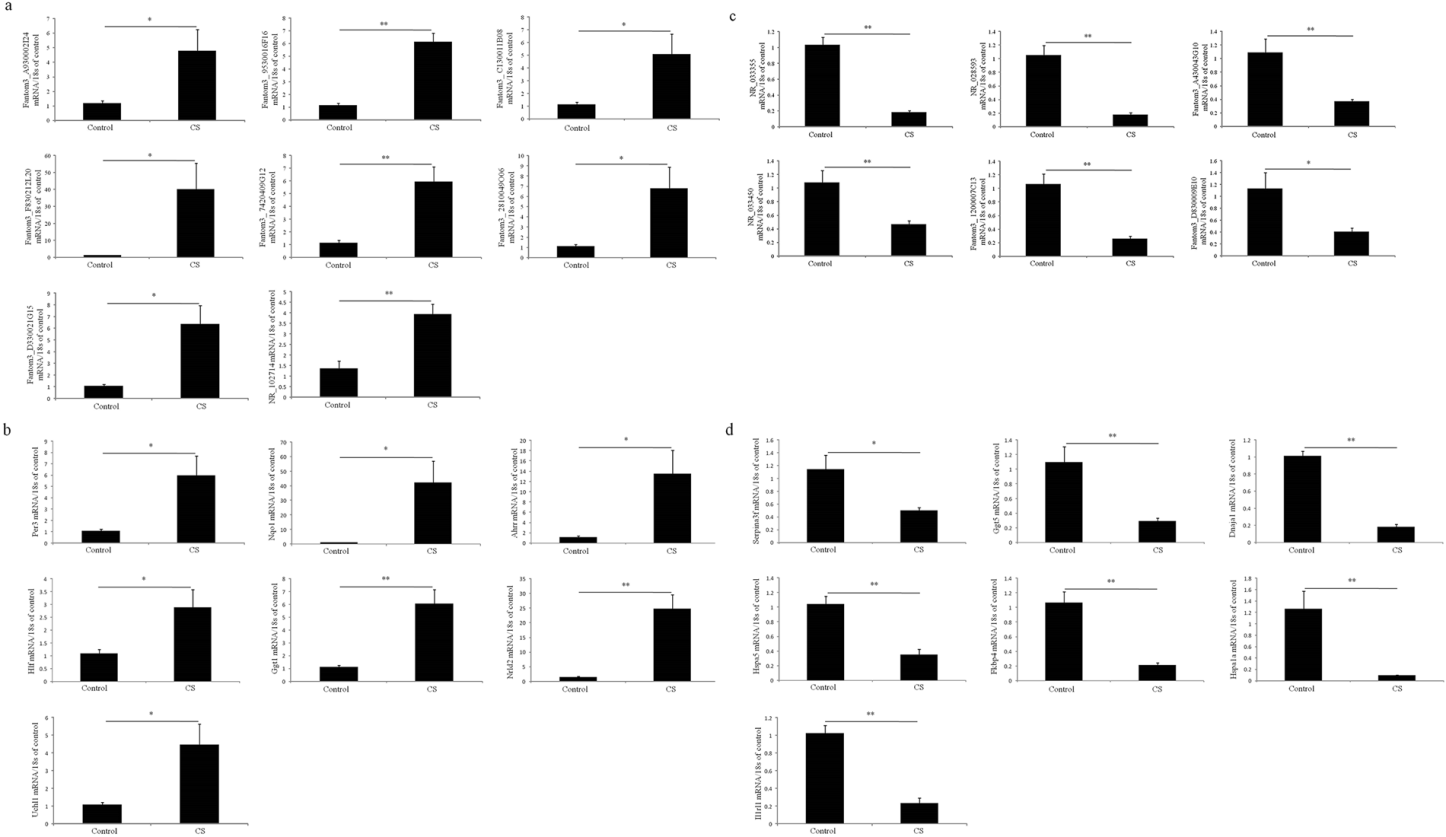
QRT-PCR validation of (**a**) Up-regulated lncRNAs, (**b**) Up-regulated mRNAs, (**c**) Down-regulated lncRNAs and (**d**) Down-regulated mRNAs in mouse (n = 8).

For practical purpose, lncRNAs, which satisfied any of the following conditions, were randomly chosen for qRT-PCR validation between other 8 chronic CS-induced COPD model mice and 8 control animals: (1) Being belonged to the top 20 significantly differential expressed lncRNAs between chronic CS-induced COPD mouse model and control animals; (2) Being belonged to the lncRNAs set, whose associated protein-coding genes were significantly differential expressed in both patients with COPD and chronic CS-induced COPD mouse model when compared to control subjects; (3) Participating in the construction of co-expression network with their associated protein-coding genes. Similarly, mRNAs, which satisfied any of the following conditions, were also randomly selected for qRT-PCR validation: (1) Being belonged to the top 20 significantly differential expressed mRNAs between chronic CS-induced COPD mouse model and control animals; (2) Being belonged to the common genes, which were significantly differential expressed both in patients with COPD and chronic CS-induced COPD mouse model when compared to control subjects; (3) Participating in the construction of co-expression network with their associated lncRNAs; (4) Being involved in the protein processing within endoplasmic reticulum pathway and taurine metabolism pathway (Both these two pathways were the significantly perturbed KEGG pathways in the present study). In this way, 3 lncRNAs (fantom3_9530016F16, NR_033355, fantom3_1200007C13) and 3 mRNAs (Ahrr, Nr1d2, Hspa1a), belonged to the top 20 significantly differential expressed lncRNAs and mRNAs respectively, were selected for qRT-PCR validation. Then, 3 lncRNAs (NR_102714, fantom3_D330021G15, fantom3_D830009E10) and their associated protein-coding genes (Uchl1, Il1rl1, Ggt5), which were significantly differential expressed both in patients with COPD and chronic CS-induced COPD mouse model, were both selected. Furthermore, 9 lncRNAs (fantom3_A930002I24, fantom3_C130011B08, fantom3_F830212L20, fantom3_7420409G12, fantom3_2810049O06, NR_028593, fantom3_A430043G10, NR_033450, fantom3_D830009E10) and their associated protein-coding genes (Hlf, Per3, Nqo1, Fkbp4, Serpina3f, Ggt5), which participated in the construction of co-expression network, were both selected. Finally, 6 differentially expressed protein-coding genes (Dnaja1, Hspa1a, Hspa5, Ggt5, and Ggt1), involved in the protein processing within endoplasmic reticulum pathway and taurine metabolism pathway, were also selected. The qRT-PCR results shown that, compared to the control animals, 8 lncRNAs (fantom3_A930002I24, fantom3_9530016F16, fantom3_C130011B08, fantom3_F830212L20, fantom3_7420409G12, fantom3_2810049O06, NR_102714, fantom3_D330021G15) and 7 mRNAs (Uchl1, Per3, Nqo1, Ahrr, Hlf, Ggt1, Nr1d2) were significantly up-regulated in lungs of chronic CS-induced COPD model mice, whereas 6 lncRNAs (NR_033355, NR_028593, fantom3_A430043G10, NR_033450, fantom3_1200007C13, fantom3_D830009E10) and 7 mRNAs (Serpina3f, Ggt5, Dnaja1, Hspa5, Fkbp4, Hspa1a, Il1rl1) were significantly down-regulated (Fig. 5).The relative expression was calculated using the 2^−△△Ct^ method. Primers lists were provided in Supplementary Tables S6, S7.

### Correlation analysis of fold change data between qRT-PCR and RNA sequencing among lncRNAs and mRNAs in mouse

For those lncRNAs and mRNAs selected for qRT-PCR validation, linear regression analyses shown that positive correlations existed in the fold change data between qRT-PCR and RNA sequencing. This outcome could verify our RNA sequencing results partly (Supplementary Figure S5).

### QRT-PCR validation of significantly differential expressed lncRNAs and mRNAs both in human bronchial epithelial cells 16HBE cells and human lung carcinoma A549 cells with and without cigarette smoke extracts (CSE) treatment

**Figure 6:**
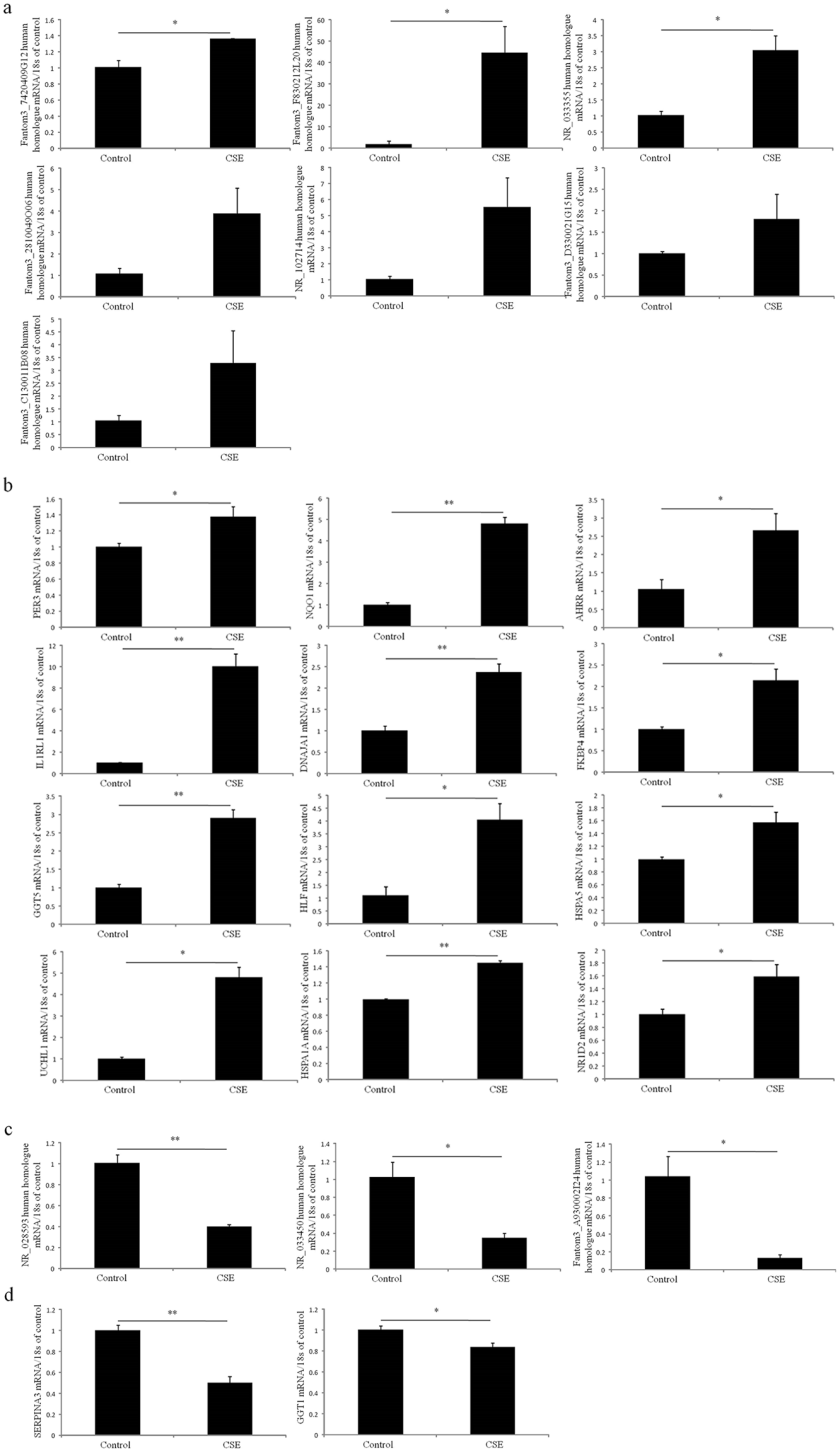
QRT-PCR validation of lncRNAs and mRNAs in 16HBE cells with and without cigarette smoke extracts (CSE) treatment. (**a**) Up-regulated lncRNAs. (**b**) Up-regulated mRNAs. (**c**) Down-regulated lncRNAs. (**d**) Down-regulated mRNAs.

A total of 11 differential expressed lncRNAs (fantom3_A930002I24, fantom3_C130011B08, Fantom3_F830212L20, fantom3_7420409G12, fantom3_2810049O06, NR_102714, fantom3_D330021G15, NR_033355, NR_028593, NR_033450, fantom3_D830009E10) were found their equivalent human homologues by blasting their sequences with the human genomic plus transcript in NCBI database^18^. Nucleotide homology of these lncRNAs and their equivalent human homologues were provided in Supplementary Table S8. QRT-PCR was performed to determine the expression patterns of these lncRNAs human homologues in both 16HBE cells and A549 cells with and without CSE treatment. The human orthologs of those significantly differential expressed mRNAs were also selected for qRT-PCR validation. Compared to the control group, 3 lncRNAs (fantom3_F830212L20, fantom3_7420409G12, NR_033355) human homologues and 12 mRNAs (UCHL1, IL1R1, DNAJA1, FKBP4, NR1D2, HSPA1A, GGT5, HLF, HSPA5, PER3, NQO1, AHRR) human orthologs were significantly up-regulated in 16HBE cells with 1% CSE treatment, whereas 3 lncRNAs (NR_028593, NR_033450, fantom3_A930002I24) human homologues and 2 mRNAs (GGT1, SERPINA3) human orthologs were significantly down-regulated (Fig. 6). Among these significantly differential expressed lncRNAs human homologues and mRNAs, 4 lncRNAs (fantom3_F830212L20, fantom3_7420409G12, NR_028593,NR_033450) human homologues and 6 mRNAs (NR1D2, PER3, HLF, AHRR, NQO1, SERPINA3) shown the common expressional tendencies in both 16HBE cells with CSE treatment and chronic CS-induced COPD mouse model when compared to control group. Although there was no significant difference in expression, 4 lncRNAs (fantom3_2810049O06, fantom3_C130011B08, NR_102714, fantom3_D330021G15) human homologues shown the same expressional tendencies in both PBMC of patients with COPD and lung tissues of chronic CS-induced COPD mouse model.

**Figure 7:**
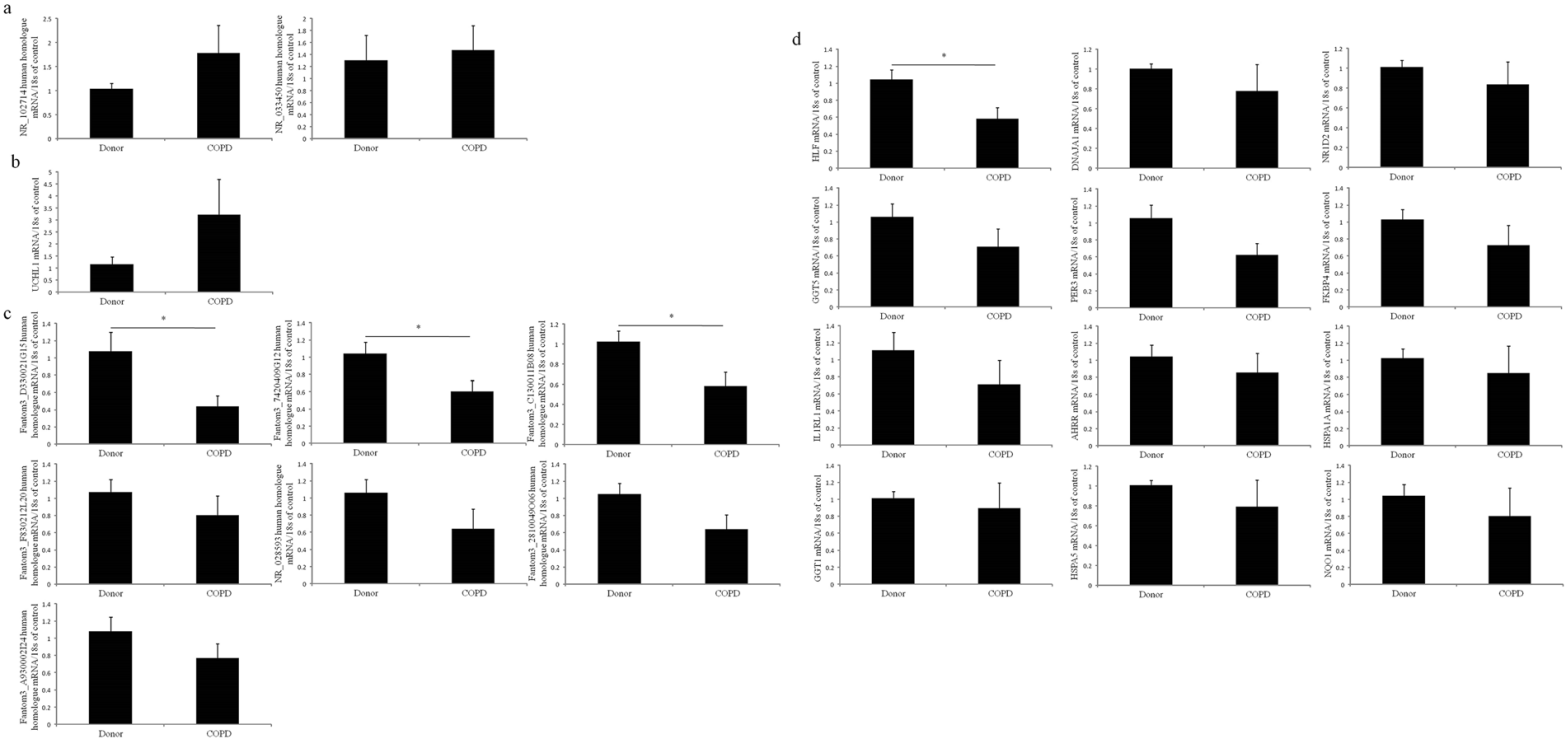
QRT-PCR validation of lncRNAs and mRNAs in A549 cells with and without CSE treatment. (**a**) Up-regulated lncRNAs. (**b**) Up-regulated mRNAs. (**c**) Down-regulated lncRNAs. (**d**) Down-regulated mRNAs.

Similarly, compared to the control group, 3 lncRNAs (fantom3_C130011B08, fantom3_7420409G12, NR_028593) and 5 mRNAs (HSPA1A, GGT1, NR1D2, NQO1, AHRR) human orthologs were significantly up-regulated in A549 cells with 1% CSE treatment, whereas 5 lncRNAs (fantom3_2810049O06, fantom3_D830009E10, NR_033450, fantom3_A930002I24, fantom3_D330021G15) human homologues and 5 mRNAs (GGT5, HLF, HSPA5, PER3, FKBP4) were significantly down-regulated (Fig. 7). Among these significantly differential expressed lncRNAs human homologues and mRNAs, 3 lncRNAs (fantom3_C130011B08, fantom3_7420409G12, fantom3_D830009E10) human homologues and 6 mRNAs (NR1D2, NQO1, AHRR, GGT5, HSPA5, FKBP4) human orthologs shown the same expressional tendencies in both 16HBE cells with CSE treatment and chronic CS-induced COPD mouse model when compared to control group.

### qRT-PCR validation of significantly differential expressed lncRNAs and mRNAs in peripheral blood mononuclear cells (PBMC) from healthy control and COPD patients

**Figure 8:**
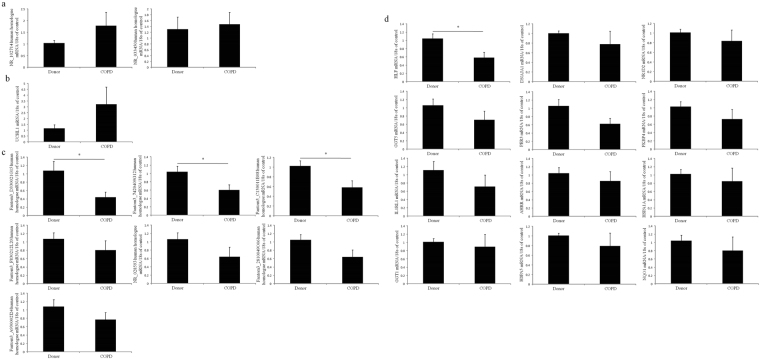
QRT-PCR validation of lncRNAs and mRNAs in peripheral blood mononuclear cells from donor and patients with COPD. Data in all bar graphs are shown as mean + standard error of donors (n = 6) and patients with COPD (n = 7). (**a**) Up-regulated lncRNAs. (**b**)Up-regulated mRNAs. (**c**) Down-regulated lncRNAs. (**d**) Down-regulated mRNAs.

The qRT-PCR results shown that, compared to the donors, 3 lncRNAs (fantom3_D330021G15, fantom3_7420409G12, fantom3_C130011B08) human homologues and HLF human ortholog were significantly down-regulated in PBMC from patients with COPD versus donors (Fig. 8). Although there was no significant difference in expression, 2 lncRNAs (NR_102714, NR_028593) human homologues and 7 mRNAs (UCHL1, DNAJA1, GGT5, FKBP4, IL1R1, HSPA1A, HSPA5) human orthologs shown the same expressional tendencies in both PBMC of patients with COPD and lung tissues of chronic CS-induced COPD mouse model.

## Discussion

For the past decades, molecular mechanisms responsible for COPD development have been widely studied. However, the knowledge about altered gene expression and subsequent biological changes involved in this disease is still insufficient. Recently, increasing evidence has shown that lncRNAs play important roles in protein-coding genes expression via cis- and/or trans- regulation mechanisms^19^. Thus, we conducted this study to better understand the roles of lncRNAs and their potential target protein-coding genes in COPD development. We used a well established chronic CS-induced COPD mouse model that imitated COPD human disease to analyze differential expression profiles of lncRNAs and mRNAs in lungs of these model mice. Hundreds of differentially expressed lncRNAs and mRNAs were found between these model mice and control animals. KEGG pathway analysis of the significantly differential expressed lncRNA associated protein-coding genes suggested that protein processing in ER was the top significantly regulated pathway in lungs of chronic CS-induced COPD model mice versus control animals. ER is a subcellular organelle, whose function is to assist protein folding and processing. Chronic CS exposure causes oxidative stress, which might induce ER stress characterized by generation of inflammatory responses, accumulation of misfolding protein and impaired lipid metabolism^20^. Moreover, chronic CS exposure activates ER stress and promotes cell death by inhibiting unfolded protein response, which serves as one of the lung cell’s physiological responses to CS-induced oxidants^21^. Within protein processing in ER pathway, several genes such as Hspa1a, Hspa1b, Hspa5 and Dnaja1 were significantly down-regulated in lungs of chronic CS-induced COPD model mice versus control animals. Most of these genes are members belonged to the heat shock protein 70 (HSP70) family, except Dnaja1 acts as a HSP70 chaperone. It has been described that HSP70 directly activates the 26 S proteasome activity, and thus reduces the accumulation of misfolding protein^22^. For example, GRP78 is a member of the HSP70 family, which has emerged as one of determinants in both processes and transportation of key lung proteins like CFTR. Loss of GRP78 leads to ER stress in lung tissues, alveolar cell apoptosis, or even neonatal death in mice^23^. These results imply that protein processing in ER pathway might be involved in the pathogenesis of chronic CS-induced COPD mouse model. Aberrant expression of genes related to HSP70 might play a role in this pathophysiologic process by activating ER stress.

In addition to protein processing in ER, another strongly perturbed KEGG pathway in our study was taurine and hypotaurine metabolism pathway. Taurine is a major intracellular free β-amino acid, which abundantly exists in many tissues of mammals. Taurine prevents cells from oxidative damage due to its potent antioxidant ability and free radical scavenging activity. Recently, taurine has been identified to have anti-inflammatory effects on LPS-induced liver injury in rat models, by inhibiting the elevations of LPS-induced aspartate transaminase and alanine transaminase; reducing the expression levels of pro-inflammatory factors, such as cyclooxygenase-2, nuclear factor κB; and decreasing the concentrations of LPS-induced inflammatory factors, including tumor necrosis factor-α and interleukin-6^24^. Moreover, taurine can attenuate both expression and activation of matrix metalloproteinases-2 and matrix metalloproteinases-9 in experimental models^25^. Similarly, hypotaurine is another critical endogenous antioxidant, which converts itself into taurine via taurine metabolic pathway. Hypotaurine has been shown to have strong antioxidant activity by preventing the inactivation of superoxide dismutase^26^. Within taurine and hypotaurine metabolism pathway, several genes such as GGT1 and GGT5, were significantly differential expressed in lungs of chronic CS-induced COPD model mice versus control animals. GGT1 is a gamma-glutamyl transferase enzyme, which is expressed in clara cells of bronchioles and alveolar type II epithelium of rat lungs, as well as normal human lung. GGT1 initiates the degradation of extracellular glutathione (GSH) by cleaveing the gamma-glutamyl bond of GSH. Thus, GGT1 has emerged as one of determinants in maintaining internal cysteine level and regulating intracellular redox status. GGT1 knockout mice with hyperoxia exposure shown severe oxidant stress in epithelium tissues, decreased GSH diffusion, bronchiolar cellular injury, and obvious pulmonary edema, suggesting that GGT1 might play a critical role in GSH homeostasis and antioxidant defense^27^. Similarly, GGT5 is another gamma-glutamyl transferase enzyme of intense interest. Overexpression of GGT5 disturbs GSH homeostasis, affects the heme oxygenase-1 level, and thus causes excessive oxidative stress^28^. These results indicate that taurine and hypotaurine metabolism pathway might also be involved in the pathogenesis of chronic CS-induced COPD mouse model. Aberrant expression of genes related to GSH metabolism like GGT1 and GGT5, might play a role in this pathophysiologic process by affecting oxidation and anti-oxidative status.

Apart from KEGG pathway analyses, chemicals enrichment analysis revealed that tobacco smoke pollution might be the major reason for the aberrant expression profiles of lncRNAs associated protein-coding genes. Within tobacco smoke pollution pathway, Nqo1 was the significantly up-regulated gene in chronic CS-induced COPD mouse model versus control animals, which also exhibited the highest fold change in our qRT-PCR validation. Nqo1 is a phase II detoxification enzyme. It mainly exists in cytoplasm and is highly expressed in many tissues, such as lung epithelium and endothelium. Nqo1 has strong antioxidant function, which results from its capability of catalyzing two electron reductions and detoxification of quinines. It has been demonstrated that Nqo1 functions as a direct superoxide reductase, assists to maintain endogenous antioxidants (e.g., ubiquinone and vitamin E) in their reduced forms, prevents or decreases the generation of free radicals and toxic reactive oxygen intermediates^29^. Loss of Nqo1 will change intracellular redox status and induce excessive ROS production^30^. Nqo1 also sustains intracellular antioxidant capacity, and thus inhibits oxidative stress mediated inflammation^31^. Recently, increasing evidence has shown that Nqo1 is intimately associated with chronic CS exposure, or may even contribute to COPD development. For example, Nqo1 is significantly higher expressed in large airway epithelium cells of smokers than that of non-smokers^32^. Functionally, Nqo1 can bind to SERPINA1 mRNA (which encodes the serine protease inhibitor α-1-antitrypsin, an important modulator of COPD) in its protein form, therefore maintaining the hepatic and serum levels of serine protease inhibitor α-1-antitrypsin, and inhibiting the activity of neutrophil elastase in mouse model^33^. These results suggest that tobacco smoke pollution might be a risk factor for the aberrant expression profiles of lncRNAs associated protein-coding genes. The disturbed expression of Nqo1, an enzyme that is strongly affected by chronic CS exposure, might aggravate the pathophysiology development of COPD in many ways, such as enhancing oxidative stress and oxidative stress mediated inflammation, and decreasing the level of serine protease inhibitor α-1-antitrypsin.

In the present study, high throughput data analysis revealed that 3 significantly differential expressed lncRNAs (NR_102714, fantom3_D330021G15 and fantom3_D830009E10) associated protein-coding genes (UCHL1,IL1RL1and GGT5) were both significantly differential expressed in patients with COPD and chronic CS-induced COPD mouse model when compared to control subjects. Furthermore, qRT-PCR validation in human bronchial epithelial cells 16HBE cells with and without CSE treatment, and in PBMC from donors and patients with COPD both shown that NR_102714 and its associated protein-coding genes UCHL1 presented the same expressional tendencies in 16HBE cells with CSE treatment, PBMC from patients with COPD and lung tissues of chronic CS-induced COPD mouse model. UCHL1 is a protein-coding gene, which is related to the degradation of unwanted, misfolded, or damaged proteins in cells. It has been described that the over expression of UCHL1 exists in more than half of the lung cancers. The result that over expression of UCHL1 exists in chronic smokers as compared with nonsmokers, is consistent with the our findings that UCHL1 is up-regulated in lungs of chronic CS-induced COPD mouse model, 16HBE cells with CSE treatment and PBMC from patients with COPD when compared to the control subjects^34^. The over expression of UCHL1 may occur in the early stage of the complex transformation from normal epithelium to malignant cells^34^. These results might suggest that lncRNA NR_102714 and its associated protein-coding genes UCHL1 play roles in the development of COPD both in mouse and human. The regulation of NR_102714 might serves as a therapeutic agent against COPD.

Taken together, our findings demonstrated that a significant number of differentially expressed lncRNAs and mRNAs were found in lungs of chronic CS-induced COPD mouse model versus control animals using RNA sequencing. These significantly differential expressed lncRNAs and their associated protein-coding genes were mainly involved in protein processing of endoplasmic reticulum pathway and taurine and hypotaurine metabolism pathway. Tobacco smoke pollution might be a major risk factor for the aberrant expression profiles of lncRNAs and their associated protein-coding genes. NR_102714 and its associated protein-coding genes UCHL1 might be involved in the development of COPD both in mouse and human. From animal model perspective, the perturbed expression profiles of lncRNAs and their associated protein-coding genes, which are both due to chronic CS exposure, might play roles in the development of COPD by causing excessive oxidative stress and ER stress. The potential regulatory link between these lncRNAs and their associated protein-coding genes might provide further clues to understand the pathogenesis of COPD.

## Methods

### Establishment of chronic CS-induced COPD mouse model

Chronic CS-induced COPD mouse model was established as we did before^35^. In brief, 12 healthy 6–8 week old and weighing 18–25 g C57BL/6J male mice were purchased from Guangdong Medical Laboratory Animal Center (Guangzhou, China). They were randomly divided into two groups. One group was housed in a smoke-free environment as control animals. Another group was given with LPS (7.5 μg per mouse in 50 μl saline; L3024; Sigma-Aldrich, St.Louis, MO) intratracheal instillation on the 0^th^ and 14^th^ day, and subsequently exposed to CS for 3 months as chronic CS-induced COPD model mice. For chronic CS-induced COPD mouse model group, mice were placed in a 60 × 57 × 100 cm fume box and exposed to 9 filter-tipped cigarettes (Red Roses from Guangdong Cigarette Factory, tar: 13 mg/cigarette; nicotine:1.3 mg/cigarette) twice a day, 6 days per week. Each CS exposure lasted about 2 hours with interval time between two CS exposures more than 4 hours. Oxygen concentration was 20–20.8%. PM 10 particle concentration was 31.37 ± 3.23 mg/m^3^. All experimental procedures were approved by the Animal Care and Use Committee of Guangzhou Medical University. All experiments were carried out according to the relevant guidelines and regulations.

### Cell culture and CSE treatment

Human bronchial epithelial cells 16HBE and human lung carcinoma A549 cells were obtained from the Cell Bank of the Chinese Academy of Sciences (Shanghai, China). Cells were cultured in DMEM supplemented with 10% fetus bovine serum (FBS), 100 units/ml penicillin and 100 ug/ml streptomycin under standard cell culture conditions.

CSE was freshly prepared on the day of the experiment. In brief, the smoke generated from two burning cigarettes (Red Roses Label; tar, 13 mg; nicotine, 1.3 mg) without filters was sucked under a constant flow rate (50 ml/10 s) into a syringe and then bubbled into a tube containing 10 ml serum-free DMEM medium. The CSE solution was sterilized using a 0.22 μm filter (Millipore, Bedford, MA, USA) and the pH was adjusted to 7.4. This CSE solution was considered 100% CSE. Then 16HBE cells and A549 cells were treated with 0% and 1%CSE concentrations for 24 hours, respectively. Cells treated with 0% and 1%CSE served as control group and CSE treatment group, respectively. There were 3 wells in each group.

### Isolation of PBMC from human subjects and the ethical approval statement

Six healthy control and seven COPD smokers were recruited from The First Affiliated Hospital of Guangzhou Medical University. PBMC samples were obtained from donors (n = 6) and patients with COPD (n = 7), respectively. Clinical characteristics of the donors and patients with COPD were provided in Supplementary Table S9. PBMC were isolated from 10 ml peripheral blood using Lymphocytes Separation Medium reagent (Dakewe, China) according to the company’s protocol. The Ethics Committee of The First Affiliated Hospital of Guangzhou Medical University approved our study. Donors and patients with COPD provide written informed consent prior to enrollment. All experiments were carried out according to the relevant guidelines and regulations.

### Pulmonary function measurement

Mouse pulmonary function was tested using the Forced Pulmonary Maneuver System (Buxco Research Systems, Wilmington, North Carolina, USA) according to the manufacturer’s protocol. In brief, mice were anesthetized with pentobarbital (50 mg/kg), tracheostomized, and then put into the body chamber of the system. The average breathing frequency was set at 150 breaths/min. Parameters of pulmonary function such as functional residual capacity (FRC), total lung capacity (TLC), chord compliance (Cchord), resistance, forced vital capacity (FVC), and forced expiration volume in 50 milliseconds (FEV50) were measured.

### BAL fluid collection, cell number counting and detection of Muc5AC in BAL fluid

Mouse BAL fluid was obtained by instilling the lung with 0.6 ml saline for 3 times. BAL fluid was collected and centrifuged at 500 g for 10 minutes. Classification and counting of cells in BAL fluid were performed with hematoxylin and eosin (H&E) staining. Muc5AC in BAL fluid was measured by ELISA according to the instructions of capture antibodies (sc-21701, Santa Cruz, CA) and detection antibodies (KPL, USA).

### Pulmonary histological analysis

Mouse lung tissues were fixed with 4% paraformaldehyde, embedded in paraffin, cut into 4 μm thick sections, and subsequently stained with H&E staining. The average alveolar intercept was assessed.

### RNA extraction

Total RNA were extracted from the mouse lung tissues and 16HBE cells by using Trizol reagent (Invitrogen, Carlsbad, CA) respectively. RNA purity was assessed using the ND-1000 Nanodrop. Each RNA sample had an A260:A280 ratio >1.8 and an A260:A230 ratio >2. RNA integrity was evaluated using the Agilent 2200 TapeStation (Agilent Technologies, USA). RNA integrity number (RIN^e^) of each RNA sample was above 7.

### Construction of RNA sequencing libraries without ribosome RNAs

For constructing RNA sequencing libraries of chronic CS-induced COPD mouse model and control animals, RNA samples from lungs of these two groups were obtained and processed. Briefly, total RNA was extracted from lungs of 3 chronic CS-induced COPD model mice and 3 control animals respectively. Ribosome RNAs were removed from total RNA using Epicentre Ribo-Zero ribosome RNAs Removal Kit (illumina, USA).RNA samples were fragmented to approximately 200 bp. Purified RNAs were reversely transcribed to the first cDNA strands. Subsequently, the second cDNA strands were synthesized, following by adaptor ligation and enrichment with a low-cycle according to the instructions of TruSeq® RNA LT/HT Sample Prep Kit (Illumina, USA). The purified library products were evaluated using the Agilent 2200 TapeStation and Qubit®2.0 (Life Technologies, USA), and diluted to 10 pM for the generation of DNA clusters. The paired-end flow cells were sequenced on HiSeq. 2500 (2 × 100 bp). Finally, high-quality RNA sequencing data were obtained and used in subsequent bioinformatics analyses after data process (e.g., removing reads without 3′ adaptors, reads without judgmental sequences, reads with 5′ adaptors contamination, reads that N-bases inside were more than 10%, and reads containing ribosome RNAs). Reads Per Kilobase of transcript per Million mapped reads (RPKM) were calculated to depict the expression levels of lncRNAs and mRNAs according to the following equation:

$${\rm{RPKM}}=\frac{{\rm{total}}\,{\rm{exon}}\,{\rm{Reads}}}{{\rm{mapped}}\,{\rm{Reads}}\,(\mathrm{millions})\ast {\rm{exon}}\,{\rm{length}}\,(\mathrm{KB})}$$

### Bioinformatics analyses of RNA sequencing data

Qualified RNA sequencing data were mapped to the mouse genome (GRCM38/mm10) and annotated using Tophat software. The expression levels of lncRNAs and mRNAs were normalized and tested using DESeq software. LncRNAs or mRNAs with fold change >2 and padj (value adjusted for multiple testing with the Benjamini-Hochberg procedure) <0.05 were defined as significantly differential expressed transcripts between chronic CS-induced COPD mouse model and control animals. Unsupervised hierarchical clustering analysis was performed to observe the different clustering patterns of lncRNAs and mRNAs between these two groups. Volcano plots analysis was performed to observe the statistic significances and differential expression levels of lncRNAs and mRNAs between these two groups.

### Prediction of significantly differential expressed lncRNAs associated protein-coding genes

Significantly differential expressed lncRNAs associated protein-coding genes were predicted via cis- and trans- regulation analyses respectively. In cis-regulation analysis, mRNAs, which were genomically neighbouring the significantly differential expressed lncRNAs with distances <10 kb, were regarded as significantly differential expressed lncRNAs associated protein-coding genes. In trans-regulation analysis, mRNAs, whose sequences matched to the sequences of significantly differential expressed lncRNAs (inclusion criteria, e < IE −5) with low complement energy (inclusion criteria, G < −20), were regarded as significantly differential expressed lncRNAs associated protein-coding genes. Then, the protein-coding genes predicted through cis- and tran-regulation analyses were overlapped with the significantly differential expressed mRNAs, with the purpose of narrowing down the field of these predicted protein-coding genes for further gene enrichment analyses and co-expression analysis.

### Enrichment analyses of the significantly differentially expressed lncRNAs potential target protein-coding genes

Both GO and KEGG pathway enrichment analyses of the significantly differential expressed lncRNAs associated protein-coding genes were performed in Database for Annotation, Visualization and Integrated Discovery (DAVID; http://david.abcc.ncifcrf.gov/) to realize the biological functions of these protein-coding genes. Chemicals enrichment analysis of these protein-coding genes was performed in Toppgene database (https://toppgene.cchmc.org/) to realize what factors might be responsible for the perturbed expression profiles of these protein-coding genes.

### Co-expression analysis between the significantly differential expressed lncRNAs and their associated protein-coding genes

Both significantly differential expressed lncRNAs and their associated protein-coding genes were selected to construct the coding-non-coding gene co-expression network by Cytoscape version 3.4 (inclusion criteria, pearson correlation coefficients >0.95). In this network, both up-regulated lncRNAs and up-regulated protein-coding genes were represented as red nodes, whereas down-regulated lncRNAs and down-regulated protein coding genes were represented as green nodes.

### High throughput data analysis between patients with COPD and chronic CS-induced COPD mouse model

High throughput data about patients with COPD were downloaded from GEO datasets (https://www.ncbi.nlm.nih.gov/geo) on March 4, 2018, and analyzed using GeneVestigator software (https://genevestigator.com/). Significantly differential expressed protein-coding genes between patients with COPD and healthy controls were obtained. Next, these significantly differential expressed genes were overlapped with those significantly differential expressed lncRNAs associated protein-coding genes, which were found between chronic CS-induced COPD mouse model and control animals in the present study, with the purpose of realizing which lncRNAs and their associated protein-coding genes might be involved in the development of COPD both in mouse model and human. The microarray data were derived from 16 studies about patients with COPD. The present study was performed according to the GEO data access policies.

### QRT-PCR

RNA was reversely transcribed to cDNA strands using PrimeScript™ RT reagent Kit with gDNA Eraser (RR047A, Takara, China). QRT-PCR was performed on CFX96–C1000 system (Bio-Rad, Hercules, CA) using SsoFast™ EvaGreen® supermix kit (Bio-Rad)0.18 s mRNA was used as an internal control. For quantitative results, expression levels of lncRNAs and mRNAs were normalized against the expression level of 18 s mRNA using the 2^−ΔΔCt^ method. Primers lists were provided in Supplementary Tables S6–S8, S10.

### Statistical analysis

Unsupervised hierarchical clustering analysis and volcano plots analysis of RNA sequencing data were performed using DESeq software. The expression levels of lncRNAs and mRNAs were calculated and represented in RPKM (Reads per kilobase of transcript per Million mapped reads). Both fold change >2 and padj <0.05 were used as threshold values to select the significantly differential expressed lncRNAs and mRNAs. Statistical analysis was analyzed with SPSS version 13.0. Linear regression analysis was performed to evaluate the correlation of fold change data between qRT-PCR and RNA sequencing. Data were analyzed using one-way ANOVA, and expressed as mean ± standard error. *P* value < 0.05 was considered statistically significant.

### Data availability statement

The datasets generated during the present study are available from the corresponding author on reasonable request.

### Ethical approval statement

All experimental procedures were approved by the Animal Care and Use Committee of Guangzhou Medical University and The Ethics Committee of The First Affiliated Hospital of Guangzhou Medical University. All experiments were carried out according to the relevant guidelines and regulations. No organs or issues were procured from prisoners.

## Electronic supplementary material

supplementary material

## References

[1] The Global Strategy for the Diagnosis, Management and Prevention of COPD, Global Initiative for Chronic Obstructive Lung Disease (GOLD) 2017. (2017).

[2] Prince, M. J. *et al*. The burden of disease in older people and implications for health policy and practice. Lancet, 10.1016/S0140-6736(14)61347-7%/ Copyright (c) 2014Elsevier Ltd. All rights reserved. (2014). 10.1016/S0140-6736(14)61347-7 25468153

[3] Roberto, R.-R. & Jørgen, V. Global initiative for chronic obstructive lung disease. *GOLD*, 1–74 (2011).

[4] Tashkin DP, Murray RP (2009). Smoking cessation in chronic obstructive pulmonary disease. Respir Med.

[5] Rahman I (2012). pharmacological antioxidant strategies as therapeutic interventions for copd. Biochim Biophys Acta.

[6] Antus, B. & Kardos, Z. Oxidative stress in COPD: molecular background and clinical monitoring. *Curr Med Chem* **22**, 627–650, CMC-64451 [pii] (2015). 10.2174/092986732205150112104411 25585265

[7] Siddiqui T (2016). Reactive oxygen species and anti-proteinases. Archives of physiology and biochemistry.

[8] Bodas M, Van Westphal C, Carpenter-Thompson R, D KM, Vij N (2016). Nicotine exposure induces bronchial epithelial cell apoptosis and senescence via ROS mediated autophagy-impairment. Free Radic Biol Med.

[9] Vu Trung, Jin Lin, Datta Pran (2016). Effect of Cigarette Smoking on Epithelial to Mesenchymal Transition (EMT) in Lung Cancer. Journal of Clinical Medicine.

[10] Rinn JL, Chang HY (2012). Genome regulation by long noncoding RNAs. Annu Rev Biochem.

[11] Li Z (2017). The degradation of EZH2 mediated by lncRNA ANCR attenuated the invasion and metastasis of breast cancer. Cell death and differentiation.

[12] Huang B (2016). Long non-coding antisense RNA KRT7-AS is activated in gastric cancers and supports cancer cell progression by increasing KRT7 expression. Oncogene.

[13] Liu B (2015). A cytoplasmic NF-kappaB interacting long noncoding RNA blocks IkappaB phosphorylation and suppresses breast cancer metastasis. Cancer cell.

[14] Pauli A (2012). Systematic identification of long noncoding RNAs expressed during zebrafish embryogenesis. Genome research.

[15] Thai Philip, Statt Sarah, Chen Ching Hsien, Liang Ellen, Campbell Caitlin, Wu Reen (2013). Characterization of a Novel Long Noncoding RNA, SCAL1, Induced by Cigarette Smoke and Elevated in Lung Cancer Cell Lines. American Journal of Respiratory Cell and Molecular Biology.

[16] Parker MM (2017). RNA sequencing identifies novel non-coding RNA and exon-specific effects associated with cigarette smoking. BMC medical genomics.

[17] Wang Q (2017). RNA sequence analysis of rat acute experimental pancreatitis with and without fatty liver: a gene expression profiling comparative study. Scientific reports.

[18] Petazzi P (2013). Dysregulation of the long non-coding RNA transcriptome in a Rett syndrome mouse model. RNA biology.

[19] Bassett AR (2014). Considerations when investigating lncRNA function *in vivo*. Elife.

[20] Cantin AM, Richter MV (2012). Cigarette smoke-induced proteostasis imbalance in obstructive lung diseases. Current molecular medicine.

[21] Lugea Aurelia, Gerloff Andreas, Su Hsin-Yuan, Xu Zhihong, Go Ariel, Hu Cheng, French Samuel W., Wilson Jeremy S., Apte Minoti V., Waldron Richard T., Pandol Stephen J. (2017). The Combination of Alcohol and Cigarette Smoke Induces Endoplasmic Reticulum Stress and Cell Death in Pancreatic Acinar Cells. Gastroenterology.

[22] Reeg S (2016). The molecular chaperone Hsp70 promotes the proteolytic removal of oxidatively damaged proteins by the proteasome. Free Radic Biol Med.

[23] Flodby P (2016). The 78-kD Glucose-Regulated Protein Regulates Endoplasmic Reticulum Homeostasis and Distal Epithelial Cell Survival during Lung Development. Am J Respir Cell Mol Biol.

[24] Kim YS (2017). Antioxidant Effect of Taurine-Rich Paroctopus dofleini Extracts Through Inhibiting ROS Production Against LPS-Induced Oxidative Stress *In Vitro* and *In Vivo* Model. Adv Exp Med Biol.

[25] Cavdar Z, Ural C, Celik A, Arslan S, Terzioglu G, Ozbal S, Yildiz S, Ergur UB, Guneli E, Camsari T, Akdogan G (2017). Protective effects of taurine against renal ischemia/reperfusion injury in rats by inhibition of gelatinases, MMP-2 and MMP-9, and p38 mitogen-activated protein kinase signaling. Biotechnic & Histochemistry.

[26] Baseggio Conrado A (2017). Carbonate Anion Radical Generated by the Peroxidase Activity of Copper-Zinc Superoxide Dismutase: Scavenging of Radical and Protection of Enzyme by Hypotaurine and Cysteine Sulfinic Acid. Adv Exp Med Biol.

[27] Jean JC (2002). Gamma-glutamyl transferase deficiency results in lung oxidant stress in normoxia. Am J Physiol Lung Cell Mol Physiol.

[28] Li W (2016). Augmented expression of gamma-glutamyl transferase 5 (GGT5) impairs testicular steroidogenesis by deregulating local oxidative stress. Cell and tissue research.

[29] Ross D, Siegel D (2017). Functions of NQO1 in Cellular Protection and CoQ10 Metabolism and its Potential Role as a Redox Sensitive Molecular Switch. Front Physiol.

[30] Dinkova-Kostova AT, Talalay P (2010). NAD(P)H:quinone acceptor oxidoreductase 1 (NQO1), a multifunctional antioxidant enzyme and exceptionally versatile cytoprotector. Archives of biochemistry and biophysics.

[31] Meyer ML (2012). NAD(P)H quinone oxidoreductase 1 regulates neutrophil elastase-induced mucous cell metaplasia. Am J Physiol Lung Cell Mol Physiol.

[32] Shahdoust M, Hajizadeh E, Mozdarani H, Chehrei A (2013). Finding genes discriminating smokers from non-smokers by applying a growing self-organizing clustering method to large airway epithelium cell microarray data. Asian Pacific journal of cancer prevention: APJCP.

[33] Di Francesco A (2016). Novel RNA-binding activity of NQO1 promotes SERPINA1 mRNA translation. Free Radic Biol Med.

[34] Carolan BJ (2006). Up-regulation of expression of the ubiquitin carboxyl-terminal hydrolase L1 gene in human airway epithelium of cigarette smokers. Cancer Res.

[35] Li D (2018). Tanshinone IIA sulfonate protects against cigarette smoke-induced COPD and down-regulation of CFTR in mice. Scientific reports.

