# Author Correction: Long non-coding RNA expression patterns in lung tissues of chronic cigarette smoke induced COPD mouse model

**DOI:** 10.1038/s41598-019-43723-4

**Published:** 2019-05-09

**Authors:** Haiyun Zhang, Dejun Sun, Defu Li, Zeguang Zheng, Jingyi Xu, Xue Liang, Chenting Zhang, Sheng Wang, Jian Wang, Wenju Lu

**Affiliations:** 1 State Key Laboratory of Respiratory Diseases, Guangzhou Institute of Respiratory Health, The First Affiliated Hospital of Guangzhou Medical University, Guangzhou, Guangdong 510180 China; 2 Division of Translational and Regenerative Medicine, Department of Medicine, The University of Arizona, Tucson, AZ 85721-0202 USA; 3 Division of Pulmonary Medicine, The People’s Hospital of Inner Mongolia, Inner Mongolia Medical University, Hohhot, Inner Mongolia China

Correction to: *Scientific Reports* 10.1038/s41598-018-25702-3, published online 15 May 2018.

This Article contains errors.

The accession codes for the raw sequencing data generated in this study were originally omitted from the Data Availability statement. The correct statement should read as follows:

**Data availability statement**

The datasets generated during the present study are available in the Short Read Archive under the accession codes SRR8616658, SRR8616659, SRR8616660, SRR8616661, SRR8616662 and SRR8616663.

