# Supplementary material for: Long non-coding RNA expression patterns in lung tissues of chronic cigarette smoke induced COPD mouse model
Source: Sci Rep. 2018 May 15;8:7609. doi: 10.1038/s41598-018-25702-3 (PMC5954018; doi:10.1038/s41598-018-25702-3)
Supplement: Supplementary file 1 — supplementary material [file 41598_2018_25702_MOESM1_ESM.pdf]

Supplement material

Long non-coding RNA expression patterns in lung tissues of chronic cigarette smoke induced COPD mouse model

Haiyun Zhang[1*], Dejun Sun[3*], Defu Li[1*], Zeguang Zheng[1], Jingyi Xu[1], Xue Liang[1], Chenting Zhang[1], Sheng Wang[1], Jian Wang[1,2 §], Wenju Lu[1 §]

[1]State Key Laboratory of Respiratory Diseases, Guangzhou Institute of Respiratory Health, The First Affiliated Hospital of Guangzhou Medical University, Guangzhou, Guangdong, 510180, China; [2]Division of Translational and Regenerative Medicine, Department of Medicine, The University of Arizona, Tucson, AZ 85721-0202, USA; [3]Division of Pulmonary Medicine, The People's Hospital of Inner Mongolia, Inner Mongolia Medical University, Hohhot, Inner Mongolia, China.

*These authors contributed equally to this work.

[§]Corresponding authors:

Wenju Lu

State Key Laboratory of Respiratory Diseases

Guangzhou Institute of Respiratory Health

The First Affiliated Hospital of Guangzhou Medical University

195 West, Dongfeng Road, Guangzhou, Guangdong, 510180

People's Republic of China

E-mail: wlu92@yahoo.com

Or

Jian Wang

State Key Laboratory of Respiratory Diseases

Guangzhou Institute of Respiratory Health

The First Affiliated Hospital of Guangzhou Medical University

195 West, Dongfeng Road, Guangzhou, Guangdong, 510180

People's Republic of China

E-mail: jianwang1986@yahoo.com

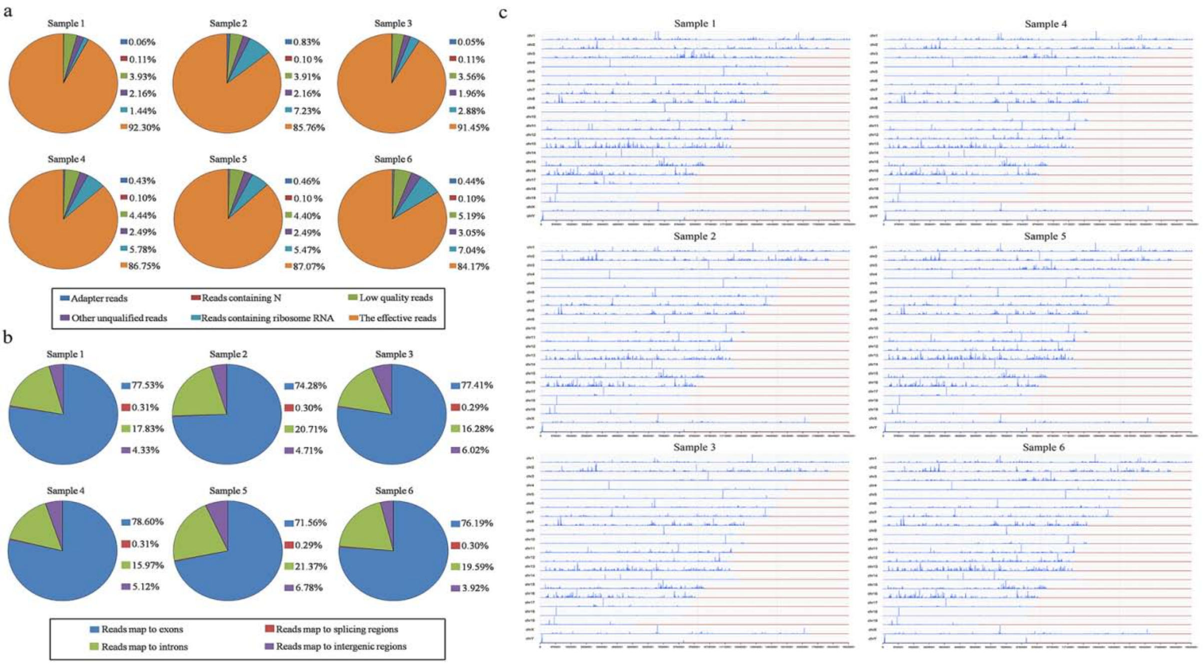

Supplementary Figure S1

Quality evaluation of RNA sequencing data. (a) Classifications of raw reads. (b) Distributions of the effective reads on the location of genes. (c) Distributions of the effective reads on the genomes. Sample1, sample2 and sample3 are belonged to control group, whereas the other samples are belonged to the chronic CS-induced COPD mouse model group.

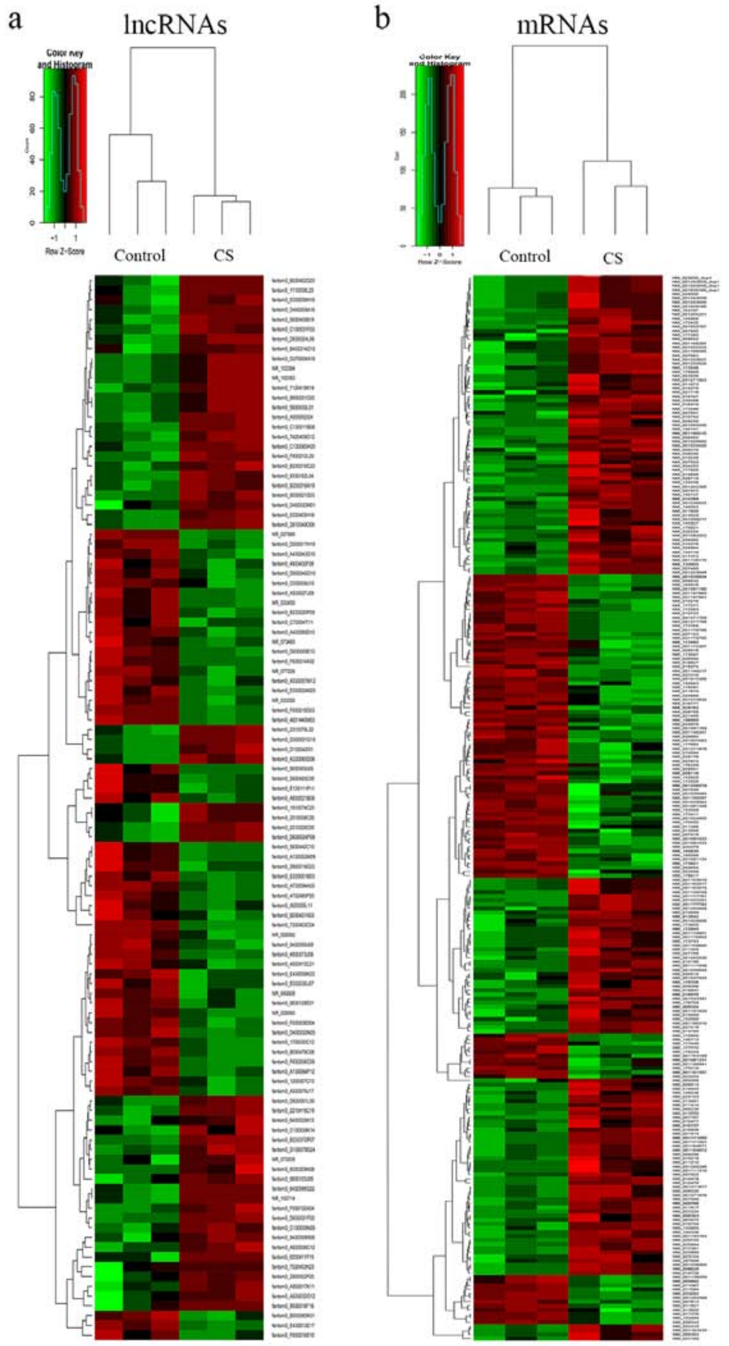

Supplementary Figure S2

Unsupervised hierarchical clustering analyses of (a) lncRNAs and (b) mRNAs in lungs of chronic CS-induced COPD model mice and control animals. RNA sequencing data are obtained from 3 mice per group. Red blocks indicate high expression levels of lncRNAs or mRNAs in each sample, whereas green blocks indicate low expression levels of lncRNAs or mRNAs.

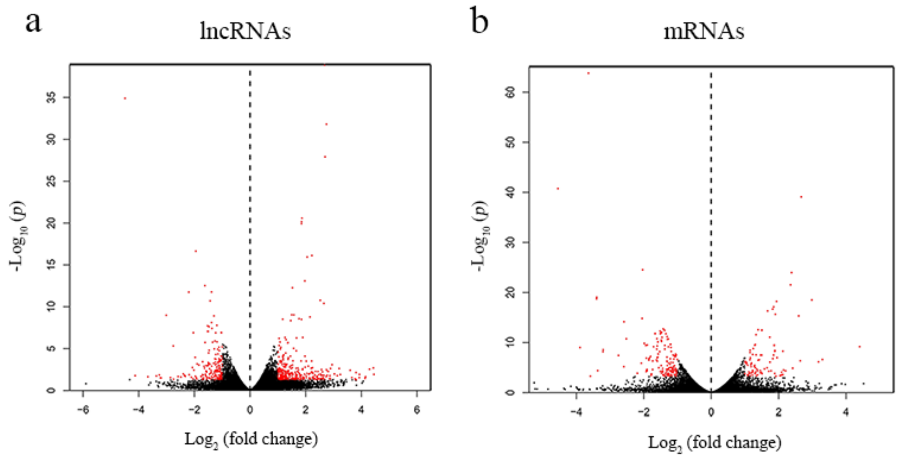

Supplementary Figure S3

Volcano plots of (a) lncRNAs and (b) mRNAs in lungs of chronic CS-induced COPD model mice and control animals. Red plots indicate significantly differential expressed lncRNAs and mRNAs between these two groups.

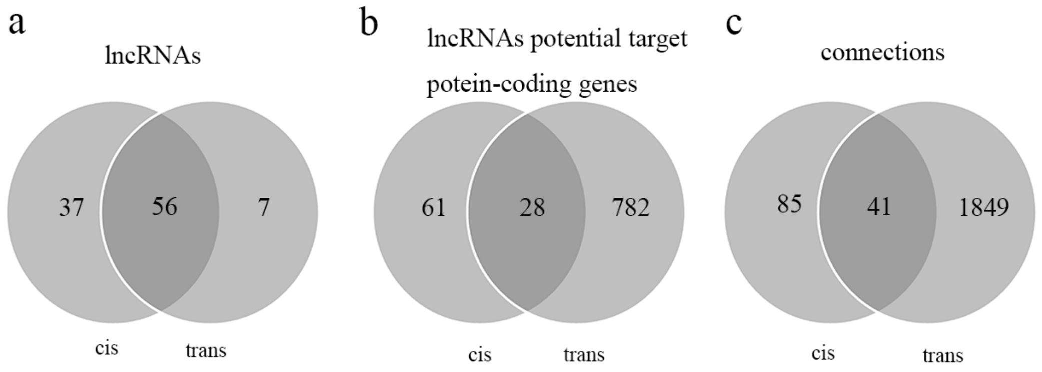

Supplementary Figure S4

Venn diagram depicting the overlaps of lncRNAs, lncRNA potential target protein coding genes, and connections between lncRNAs and their associated protein coding genes in lncRNA cis- and trans- regulation patterns.(a) Overlap of lncRNAs.(b) Overlap of lncRNAs potential target protein-coding genes.(c) Overlap of connections between lncRNAs and their potential target protein coding genes.

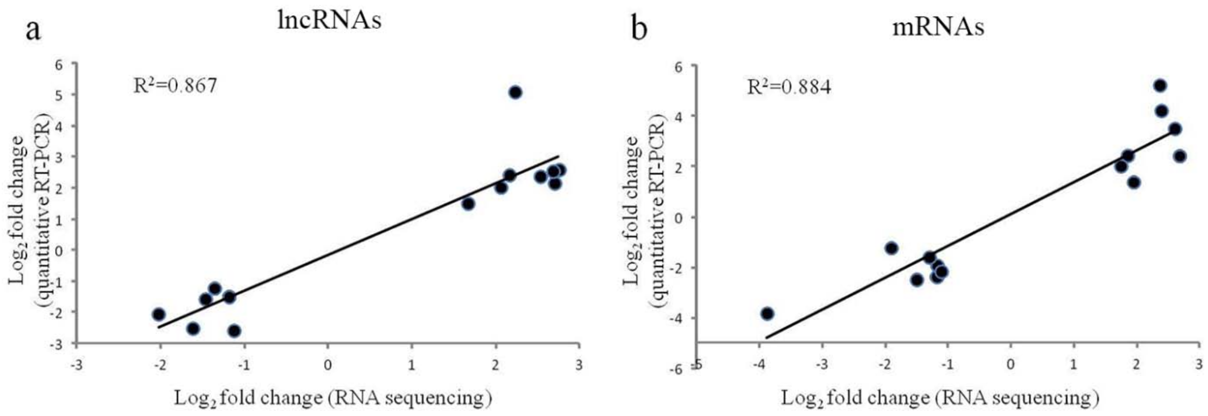

Supplementary Figure S5

Correlation analysis of fold change data between qRT-PCR and RNA sequencing in mouse. (a) Up- and down- regulated lncRNAs selected for qRT-PCR validation. (b) Up- and down- regulated mRNAs selected for qRT-PCR validation. Black plots indicate lncRNAs or mRNAs. R represents correlation coefficient.

Supplementary Table S1

RNA sequencing reads of chronic CS-induced COPD mouse model and control animals.

ID	Group	Raw reads		Total raw reads	Reads with adapters		Total reads with adapters	Reads containing N		Total reads containing N	Low quality reads		Total low quality reads	Other reads needed to be removed from analysis*	Reads containing ribosome RNA	The effective reads
		Read1	Read2		Read1	Read2		Read1	Read2		Read1	Read2				
Sample 1	Control animals	80093321	80093321	'160,186,642	46,697	46,453	'93,150	78558	89768	'168,326	2215700	4083721	'6,299,421	'3,462,719	'2,310,630	'147,852,396
Sample 2	Control animals	72307427	72307427	'144,614,854	606340	599237	'1,205,577	70386	80520	'150,906	2038025	3616448	'5,654,473	'3,129,488	'10,451,530	'124,022,880
Sample 3	Control animals	69158955	69158955	'138,317,910	36624	36368	'72,992	68331	77775	'146,106	1888339	3030150	'4,918,489	'2,708,761	'3,983,556	'126,488,006
Sample 4	Chronic CS-induced COPD mouse model	77890883	77890883	'155,781,766	342395	334134	'676,529	76107	86940	'163,047	2215125	4706696	'6,921,821	'3,876,319	'8,998,894	'135,145,156
Sample 5	Chronic CS-induced COPD mouse model	84100609	84100609	'168,201,218	392899	382975	'775,874	82431	93808	'176,239	2386172	5013807	'7,399,979	'4,186,832	'9,203,034	'146,459,260
Sample 6	Chronic CS-induced COPD mouse model	71413600	71413600	'142,827,200	317908	310309	'628,217	69695	79871	'149,566	2035508	5376206	'7,411,714	'4,358,709	'10,060,178	'120,218,816

*Other reads needed to be removed from analysis, such as reads without 3' adapter reads without judgment sequences, reads with 5' adapter contamination and so on.

Supplementary Table S2

RNA sequencing data analysis: mapping of RNA sequencing reads to exons, introns, splicing regions and intergenic regions in chronic CS-induced COPD mouse model and control animals.

ID	Group	The effective reads	Total mapped reads	Reads map to exons	Reads map to splicing regions	Reads map to introns	Reads map to intergenic regions
Sample 1	Control animals	147,852,396	'140,574,061	'108,993,577	'433,245	'25,065,773	'6,081,466
Sample 2	Control animals	'124,022,880	'115,836,570	'86,041,686	'346,303	'23,992,414	'5,456,167
Sample 3	Control animals	'126,488,006	'119,128,122	'92,205,678	'350,935	'19,394,919	'7,176,590
Sample 4	Chronic CS-induced COPD mouse model	'135,145,156	'125,783,965	'98,870,957	'385,381	'20,087,387	'6,440,240
Sample 5	Chronic CS-induced COPD mouse model	'146,459,260	'136,097,519	'97,391,052	'389,428	'29,087,258	'9,229,781
Sample 6	Chronic CS-induced COPD mouse model	'120,218,816	'112,272,198	'85,544,144	'331,616	'21,997,497	'4,398,941

Supplementary Table S3

Top 20 significantly up- and down- regulated lncRNAs in lungs of chronic CS-induced COPD mouse model compared with control animals.

Gene	Transcript	Position	Strand	Fold Change	*P* Value
**Up-regulated**					
Fantom3_2810049O06	AK012936	chr4:151003658-151004621	—	6.72	＜0.001
Fantom3_C130011B08	AK081361	chr4:151042408-151043788	—	6.49	＜0.001
Fantom3_D330021G15	AK084610	chr4:151011436-151031497	—	6.40	＜0.001
Fantom3_5330405H16	AK030382	chr4:151033075-151036561	—	6.29	＜0.001
Fantom3_7420409G12	AK135741	chr4:151018475-151021895	+	5.75	＜0.001
Fantom3_F830212L20	AK157369	chr8:107385117-107388684	+	4.67	＜0.001
Fantom3_2900002P20	AK013478	chr5:120940505-120941428	—	4.64	0.035
Fantom3_9530016F16	AK035325	chr1:127983186-127984687	—	4.46	＜0.001
Fantom3_A930002I24	AK044236	chr11:90336537-90338283	—	4.15	＜0.001
Fantom3_A630036C12	AK041770	chrX:162628954-162629652	—	4.06	0.036
Fantom3_6230411F15	AK134477	chr6:126708330-126736811	—	3.93	0.013
Fantom3_C130085H20	AK081893	chr7:110120216-110121740	—	3.91	＜0.001
Fantom3_A630032O12	AK041731	chr1:127982282-127983640	—	3.72	＜0.001
Fantom3_D630024P09	AK085437	chr15:81825471-81826859	+	3.64	＜0.001
Fantom3_2310028D20	AK009518	chr15:81825121-81826859	+	3.62	＜0.001
Fantom3_2310076L22	AK010205	chr15:81826045-81826859	+	3.61	＜0.001
Fantom3_B230316C23	AK045874	chr11:90337983-90339337	—	3.61	＜0.001
Fantom3_9430099M06	AK020530	chr8:102706048-102706790	—	3.56	0.034
Fantom3_9930123J05	AK037130	chr1:127978904-127981691	—	3.39	＜0.001
Fantom3_D630021F02	AK085396	chr11:95961598-95964859	—	3.19	＜0.001
**Down-regulated**					
Fantom3_7330403C04	AK078618	chr17:34957618-34959819	+	0.04	＜0.001
Fantom3_E430013E17	AK088332	chr7:113219369-113220523	+	0.12	＜0.001
Fantom3_4732480P20	AK076384	chr2:126600233-126618661	—	0.15	0.001
Fantom3_A530079J17	AK080197	chr5:134738232-134738818	—	0.22	＜0.001
Fantom3_1200007C13	AK004627	chr2:167827692-167833646	—	0.24	＜0.001
Fantom3_D430030N05	AK052469	chr11:58202414-58204772	+	0.25	0.016
Fantom3_F830218D03	AK172595	chr6:29853890-29867419	+	0.26	＜0.001

Fantom3_B930085K01	AK081095	chr11:43561869-43564424	＋	0.31	0.039
NR_033355	NR_033355	chr4:43730033-43734534	＋	0.32	＜0.001
Fantom3_F830208D04	AK157250	chr11:20461258-20464664	－	0.33	0.001
Fantom3_8030479C08	AK033265	chr15:101931536-101933987	＋	0.35	＜0.001
Fantom3_E030001B03	AK086777	chr5:134744725-134747253	－	0.35	＜0.001
Fantom3_E030024M20	AK087068	chr5:134725602-134729106	－	0.35	＜0.001
Fantom3_2900019G23	AK132033	chr2:23012822-23014153	－	0.36	0.007
Fantom3_1700030C12	AK006530	chr11:23497756-23499640	＋	0.36	＜0.001
Fantom3_A430043G10	AK040013	chr12:104247895-104248893	＋	0.36	0.004
Fantom3_F630314A02	AK155529	chr5:43784045-43786115	－	0.37	＜0.001
Fantom3_A730094A05	AK139286	chr4:40720058-40722867	－	0.38	＜0.001
Fantom3_A430085D10	AK079839	chr6:29876678-29879068	＋	0.38	＜0.001
NR_033450	NR_033450	chr12:104247895-104254405	＋	0.39	＜0.001

Supplementary Table S4

Top 20 significantly up- and down- regulated mRNAs in lungs of chronic CS-induced COPD mouse model compared with control animals.

Gene symbol	Gene Name	Transcript	Position	Strand	Fold Change	*P* Value
**Up-regulated**						
Dbp	D site albumin promoter binding protein	NM_016974	chr7:45705247-45710203	＋	21.16	＜0.001
Ces1g	carboxylesterase 1G	NM_021456	chr8:93302369-93337209	—	9.83	＜0.001
Cyp2a5	cytochrome P450, family 2, subfamily a, polypeptide 5	NM_007812	chr7:26835339-26843264	＋	9.07	＜0.001
Necab1	N-terminal EF-hand calcium binding protein 1	NM_178617	chr4:14952245-15149131	—	7.91	＜0.001
Per3	period circadian clock 3	NM_011067	chr4:151003655-151044622	—	6.37	＜0.001
Olfr1342	olfactory receptor 1342	NM_146713	chr4:118689503-118690450	—	6.25	＜0.001
Ahrr	aryl-hydrocarbon receptor repressor	NM_009644	chr13:74211118-74292309	—	6.04	＜0.001
Bpifc	BPI fold containing family C	NM_177772	chr10:85959691-86011860	—	5.33	0.003
Nr1d2	nuclear receptor subfamily 1, group D, member 2	NM_011584	chr14:18204056-18239106	—	5.21	＜0.001
Nqo1	NAD(P)H dehydrogenase, quinone 1	NM_008706	chr8:107388225-107403205	—	5.11	＜0.001
Mogat2	monoacylglycerol O-acyltransferase 2	NM_177448	chr7:99219084-99238611	—	4.61	0.008
Enthd1	ENTH domain containing 1	NM_001163189	chr15:80452240-80560470	—	4.37	0.032
Npy	neuropeptide Y	NM_023456	chr6:49822729-49829505	＋	4.36	＜0.001
Tcfl5	transcription factor-like 5 (basic helix-loop-helix)	NM_178254	chr2:180621957-180642691	—	4.27	0.033
Oxct2a	3-oxoacid CoA transferase 2A	NM_022033	chr4:123321875-123323679	—	4.25	0.014
D7Ertd443e	DNA segment, Chr 7, ERATO Doi 443, expressed	NM_001081331	chr7:134266262-134376828	—	4.16	0.037
Spag11b	sperm associated antigen 11B	NM_001039563	chr8:19140759-19143007	＋	3.87	＜0.001

Hlf	hepatic leukemia factor	NM_172563	chr11:90336535-90390917	—	3.84	＜0.001
Sult1d1	sulfotransferase family 1D, member 1	NM_016771	chr5:87554650-87569006	—	3.78	0.049
Igfbp2	insulin-like growth factor binding protein 2	NM_008342	chr1:72824480-72852471	＋	3.74	＜0.001
**Down-regulated**						
Hspa1b	heat shock protein 1B	NM_010478	chr17:34956429-34959238	—	0.04	＜0.001
Hspa1a	heat shock protein 1A	NM_010479	chr17:34969359-34972156	—	0.07	＜0.001
Spon2	spondin 2, extracellular matrix protein	NM_133903	chr5:33213518-33218238	—	0.08	＜0.001
Gbp10	guanylate-binding protein 10	NM_001039646	chr5:105215699-105239533	—	0.08	0.049
Arntl	aryl hydrocarbon receptor nuclear translocator-like	NM_007489	chr7:113207465-113314126	＋	0.09	＜0.001
Mucl1	mucin-like 1	NM_009268	chr15:103751923-103899300	—	0.10	0.008
Fga	fibrinogen alpha chain	NM_001111048	chr3:83026153-83033617	＋	0.11	＜0.001
Npas2	neuronal PAS domain protein 2	NM_008719	chr1:39194272-39363240	＋	0.15	＜0.001
Nfil3	nuclear factor, interleukin 3, regulated	NM_017373	chr13:52967209-52981039	—	0.17	0.002
Avpr1a	arginine vasopressin receptor 1A	NM_016847	chr10:122448499-122453453	＋	0.17	＜0.001
Gm12250	predicted gene 12250	NM_001135115	chr11:58183843-58190198	＋	0.17	＜0.001
Gbp6	guanylate binding protein 6	NM_194336	chr5:105270702-105293699	—	0.22	0.020
Hdc	histidine decarboxylase	NM_008230	chr2:126593659-126624024	—	0.24	＜0.001
Cd209a	CD209a antigen	NM_133238	chr8:3743395-3748984	—	0.24	＜0.001
Hsph1	heat shock 105kDa/110kDa protein 1	NM_013559	chr5:149616845-149636315	—	0.24	＜0.001
Fam124b	family with sequence similarity 124, member B	NM_173425	chr1:80198699-80213944	—	0.25	＜0.001
Ccnjl	cyclin J-like	NM_001045530	chr11:43528749-43586999	＋	0.26	＜0.001
Serpina3f	serine (or cysteine) peptidase inhibitor, clade A, member 3F	NM_001168294	chr12:104214544-104221129	＋	0.26	0.007

Tgtp2	T cell specific GTPase 2	NM_001145164	chr11:49057196-49064212	—	0.26	＜0.001
Klra7	killer cell lectin-like receptor, subfamily A, member 7	NM_014194	chr6:130218605-130231687	—	0.27	0.005

Supplementary Table S5

High throughput studies about patients with COPD from GEO datasets.

Experiment ID in GEO database	Authors	Study design	Study type	Organism	Platform	Samples
GSE475	Rubinstein NA	case control study	in vivo	Homo sapiens	HS_AFFY_U133A[*]	7
GSE10006	Carolan B et al. / Crystal RG	case control study	in vivo	Homo sapiens	HS_AFFY_U133PLUS_2[*]	84
GSE22148	Singh D et al. / Celli B	clinical history study	in vivo	Homo sapiens	HS_AFFY_U133PLUS_2	136
GSE13896	Shaykhiev R et al. / Crystal RG	case control study	in vivo	Homo sapiens	HS_AFFY_U133PLUS_2	46
GSE42057	Bowler RP et al. / Hughes GJ	case control study, clinical history study	in vivo	Homo sapiens	HS_AFFY_U133PLUS_2	131
GSE47460	Tedrow J et al. / Schwartz DA	case control study	in vivo	Homo sapiens	HS_AGIL_4x44K[#]	151
GSE47460	Tedrow J et al. / Schwartz DA	case control study, clinical history study	in vivo	Homo sapiens	HS_AGIL_8x60K[§]	424
GSE22047	Butler MW et al. / Crystal RG	case control study	in vivo	Homo sapiens	HS_AFFY_U133PLUS_2	125
GSE8581	Bhattacharya S et al. / Mariani TJ	clinical history study	in vivo	Homo sapiens	HS_AFFY_U133PLUS_2	53
GSE11784	Tilley AE et al. / Crystal RG	case control study	in vivo	Homo sapiens	HS_AFFY_U133PLUS_2	110

GSE20257	Shaykhiev R et al. / Crystal RG	case control study	in vivo	Homo sapiens	HS_AFFY_U133PLUS_2	76
GSE29133	Fujino N / Kubo H	case control study	in vivo	Homo sapiens	HS_AFFY_U133PLUS_2	6
GSE37768	Bastos R et al. / Kalko SG	case control study	in vivo	Homo sapiens	HS_AFFY_U133PLUS_2	37
GSE56341	Vucic EA	case control study	in vivo	Homo sapiens	HS_AFFY_ST1_0[※]	22
GSE37147	Steiling K / Spira A	case control study	in vivo	Homo sapiens	HS_AFFY_ST1_0	244
GSE57148	Kim WJ et al. / Lee SD	case control study, clinical history study	in vivo	Homo sapiens	HS_mRNASeq_HUMAN_GL	165

[*]HS_AFFY_U133A: Affymetrix Human Genome U133A Array.

[*]HS_AFFY_U133PLUS_2: Affymetrix Human Genome U133 Plus 2.0 Array.

[#]HS_AGIL_4x44K: Agilent-014850 Whole Human Genome Array 4x44K G4112F.

[§]HS_AGIL_8x60K: Agilent-028004 SurePrint G3 Human GE 8x60K Array.

[※]HS_AFFY_ST1_0: Affymetrix Human Gene 1.0 ST Array.

Supplementary Table S6

Primers used for quantitative RT-PCR validation of 6 up-and down-regulated lncRNAs in mouse.

Gene name	Tran script	Forward (5'→3')	Reverse (5'→3')	Product length (bp)	Tm (℃)
**Up-regulated**					
Fantom3_A930002I24	AK044236	TTCAACTCCAGGTGTTCGGT	CCCCAACACAGATTTTGGCA	122	60
Fantom3_9530016F16	AK035325	CCTGGTCTGTTGTGGTGAGA	CAAGTCCCACCCACCTCTAG	192	60
Fantom3_C130011B08	AK081361	AAGCCCAGTACCCTAGATGC	TTCCACTGCACATGAGGACT	115	60
Fantom3_F830212L20	AK157369	TTGGAATGCTGAGACCCTGT	GGAGTGAAAACACGTGGCTT	173	60
Fantom3_7420409G12	AK135741	TCTGCTGATAGGACGAGCTG	TTCTCACTAGCTGCCGAACA	163	60
Fantom3_2810049O06	AK012936	TGAAGTGATTTGGGAGGCCT	AGCAGGAACCAGAGACCATC	112	60
NR_102714	NR_102714	GCAGAGAGGGAGACCATCAG	TGTAGCGTACCATCCATCCA	145	60
Fantom3_D330021G15	AK084610	CCTGTGTTCAAGGGTCCACT	ACAGGTGCTGGCAACTTCTT	88	60
**Down-regulated**					
NR_033355	NR_033355	CAGAAATCATGGAGACCGCG	GATGCAGGTAATGGCCATGG	73	60
NR_028593	NR_028593	AGGGTACTGGAGTGGTTTGG	GGCCATCAACCAGAAAAGCA	75	60
Fantom3_A430043G10	AK040013	GGACCCCACTTCCCTTGTAA	AGTGTCTGAGCAGGTGTCTC	89	60
NR_033450	NR_033450	GCATCTGAAACACTTGACGCC	CCATGCATTTGCAGGGTTGAG	136	60
Fantom3_12000	AK0	TACAAACCTGGGC	AAGCAGAGGC	103	60

07C13	04627	TCACCAC	TAACGGTGTC		
Fantom3_D830009E10	AK085788	TGGTCTATACCCGCTGACCT	CTTTCCTCAGCGAGGGATGT	108	60
**Internal control**					
18s	18s	GCAATTATTCCCCATGAACG	GGCCTCACTAAACCATCCAA	123	60

Supplementary Table S7

Primers used for quantitative RT-PCR validation of 6 up-and down-regulated mRNAs in mouse.

Gene name	Transcript	Forward (5'→3')	Reverse (5'→3')	Product length (bp)	Tm (℃)
**Up-regulated**					
Per3	NM_011067	GAAGCAGTGCCAGCAGTATA	AAGGGCTTCGTCTCTCTGTC	101	60
Nqo1	NM_008706	GGAAGCTGCAGACCTGGTGA	CCTTTCAGAATGGCTGGCA	69	60
Hlf	NM_172563	GACAGCTCCCCTTGAACCC	CTGCTGCTCTCATCGTCCA	151	61
Ggt1	NM_008116	TTTGTCATCATCGGCCTCTGT	CCCGTCCAATCTCTGAGCAG	115	61
Ahrr	NM_009644	GTGGTTACGATGGACTCAAGG	GTCCCCTGAACAGTGAAATGC	124	60
Nr1d2	NM_011584	TGAACGCAGGAGGTGTGATTG	GAGGACTGGAAGCTATTCTCAGA	100	62
Uchl1	NM_011670	AGGGACAGGAAGTTAGCCCTA	AGCTTCTCCGTTTCAGACAGA	160	60
**Down-regulated**					
Serpina3f	NM_001168294	TGGGAGATGCCCTTTGATCC	AGTAGGGTGTCGTCAGGTTATTA	112	60
Ggt5	NM_011820	TTCAATGGGACAGAAACCTTGAG	TCCCTGTGTATAAGACCTCCG	109	61
Dnaja1	NM_008298	ACCACTTACTACGATGTTTTGGG	GCCCTCTTTAATCGCCTGCT	213	61
Hspa5	NM_001163434	GCATCACGCCGTCGTATGT	ATTCCAAGTGCGTCCGATGAG	134	61
Fkbp4	NM_010219	CCTCTCGAAGGAGTGGACATC	TCCCCGATCATGGGTGTCT	98	61
Hspa1a	NM_010479	TGGTGCAGTCCGACATGAAG	GCTGAGAGTCGTTGAAGTAGGC	219	61
Il1rl1	NM_001025602	TGACACCTTACAAAACCCGGA	AGGTCTCTCCCATAAATGCACA	178	61
**Internal control**					
18s	18s	GCAATTATTCCCCATGAACG	GGCCTCACTAAACCATCCAA	123	60

Supplementary Table S8

Nucleotide homology, gene loci and primers used for quantitative RT-PCR validation of human homologue of the differential expressed lncRNAs in chronic CS-induced COPD mouse model versus control animals.

The equivalent lncRNAs in human	Nucleotide homology between mouse and human (%)	Gene loci	Strand	Forward (5'→3')	Reverse (5'→3')	Product length (bp)	Tm (℃)
Fantom3A930002I24 human homologue	80	Chr17: 55324820-55325059	＋	TTTTGGCAACACCACAAAGA	GATAAAGGTTTGGACTTAACAGCA	116	60
Fantom3_C130011B08 human homologue	83	Chr1: 7785438-7785587	+	TGAACAGCAAGATCGAAACAG	CACAGCGGAGAGCATAGTTG	131	60
Fantom3_F830212L20 human homologue	73	chr16: 69710466-69710627	—	TATGGCAGAAGGGAATTGCT	GCTTTGTAATTGAAAGCAAGAAA	162	60
Fantom3_7420409G12 human homologue	88	chr1: 7819372-7819436	+	GCACAGCCCATCCTATCAAC	TTGCGGTCTCATACCTGATG	62	60
Fantom3_2810049O06 human homologue	66	chr1: 7844617-7845152	+	TTCTTTGCCTTAGCACACGTT	CTGACAGTTCTGCGACAAGC	82	60
NR_102714 human homologue	83	chr4: 41256890-41257009	+	CCGCTAGCTGTTTTTCGTCT	GTTGATCTCCATCGGCTTGA	120	60
Fantom3_D330021G15 human homologue	81	Chr1: 7803047-7819420	+	ATTGGTCGGCATAAAGTTCG	GCTCACGTGAACTGGCTGTA	144	60

NR_033355 human homologue	78	Chr9: 35911116-35911574	+	GCCCTGAAATTCTGAAACCA	CAGATTTGGAAAGGCCCATA	97	60
NR_028593 human homologue	81	Chr12: 9233455-9233555	—	GAGGACCTGTCTGTGGCATC	AGACCTGAGTGGAGGAGAAGC	101	60
NR_033450 human analogues	72	Chr14: 94614589-94623819	+	CGTGGTGGAGCTGAAGTACA	GAGTCTCTCCACCGCTTCAG	120	60
Fantom3_D830009E10 human homologue	81	Chr22: 24232825-24244755	—	CTCCAAGGTCTGCTCGGATA	ATGCTCTGAGGGTTGACGAC	111	60

Supplementary Table S9

Clinical characteristics of the donors and patients with COPD. The age variable of two groups fits normal distribution and homogeneity of variance.

No.	Sex	Age	Smoke	Underlying Condition	FEV_1/FVC (%)
1	Male	90	No	Donor	＞70
2	Male	80	No	Donor	＞70
3	Male	52	No	Donor	＞70
4	Male	77	No	Donor	＞70
5	Male	61	No	Donor	＞70
6	Male	67	No	Donor	＞70
7	Male	68	Yes	COPD	＜70
8	Male	55	Yes	COPD	＜70
9	Male	68	Yes	COPD	＜70
10	Male	63	Yes	COPD	＜70
11	Male	60	Yes	COPD	＜70
12	Male	73	Yes	COPD	＜70
13	Male	74	Yes	COPD	＜70

Supplementary Table S10

Primers used for quantitative RT-PCR validation of significantly differential expressed mRNAs in human bronchial epithelial cells 16HBE cells, human lung carcinoma A549 cells and human PBMC.

Gene name	Transcript	Forward(5'→3')	Reverse(5'→3')	Product length (bp)	Tm (℃)
PER3	NM_016831	GCAGAGGAAATTGGCGGACA	GGTTTATTGCGTCTCTCCGAG	123	60
NQO1	NM_001025433	GAAGAGCACTGATCGTACTGGC	GGATACTGAAAGTTCGCAGGG	196	60
HLF	NM_002126	CTGGGGCCTACCTTATGGGA	GGGGAATGCCATTTTCTGACA	103	60
GGT1	NM_001288833	TTCATCGCTGTGGTGCAAGC	TCTGCTGCTCACAGGGGAAG	194	60
AHRR	NM_001242412	CCCCGCCCTTGGAGACAGGA	AGTACTCGGTGGGCGTGCCT	87	60
NR1D2	NM_001145425	TTTAGTGGCATGGTTCTACTGTG	AGCCTTCGCAAGCATGAACT	82	60
UCHL1	NM_004181	AATGTCGGGTAGATGACAAGGT	GGCATTCGTCCATCAAGTTCATA	88	60
SERPINA3	NM_001085	TGCCAGCGCACTCTTCATC	TGTCGTTCAGGTTATAGTCCCTC	167	60
GGT5	NM_004121	GTCAGCCTAGTCCTGCTGG	GGATGGCTCGTCCAATATCCG	157	60
DNAJA1	NM_001539	ACTGGAGCCAGGCGATATTAT	CTTCAACGAGCTGTATGTCCAT	107	60
HSPA5	NM_005347	CATCACGCCGTCCTATGTCG	CGTCAAAGACCGTGTTCTCG	104	60
FKBP4	NM_002014	GAAGGCGTGCTGAAGGTCAT	TGCCATCTAATAGCCAGCCAG	103	62
HSPA1A	NM_005345	GAACAAGCGAGCCGTGAG	AGGGTGCTTCGGAACAGG	177	60
IL1RL1	NM_003856	ATGGGGTTTTGGATCTTAGCAAT	CACGGTGTAACTAGGTTTTCCTT	138	60
**Internal control**					
18S	18S	GCAATTATTCCCCATGAACG	GGCCTCACTAAACCATCCAA	123	60
